# COVID-19 vaccine strategies for Aotearoa New Zealand: a mathematical modelling study

**DOI:** 10.1016/j.lanwpc.2021.100256

**Published:** 2021-08-19

**Authors:** Trung Nguyen, Mehnaz Adnan, Binh P Nguyen, Joep de Ligt, Jemma L Geoghegan, Richard Dean, Sarah Jefferies, Michael G Baker, Winston KG Seah, Andrew A Sporle, Nigel Peter French, David R Murdoch, David Welch, Colin R Simpson

**Affiliations:** 1 Institute of Environmental Science and Research, New Zealand; 2 School of Mathematics and Statistics, Victoria University of Wellington, New Zealand; 3 Department of Microbiology and Immunology, University of Otago, New Zealand and Institute of Environmental Science and Research, New Zealand; 4 Department of Public Health, University of Otago, New Zealand; 5 School of Engineering and Computer Science, Victoria University of Wellington, New Zealand; 6 Department of Statistics, The University of Auckland, New Zealand and iNZight Analytics Ltd; 7 School of Veterinary Science, Massey University, New Zealand; 8 Department of Pathology and Biomedical Science, University of Otago, New Zealand; 9 School of Computer Science, The University of Auckland, New Zealand; 10 School of Health, Wellington Faculty of Health, Victoria University of Wellington, Wellington, New Zealand; 11 Usher Institute, The University of Edinburgh, Edinburgh, United Kingdom

**Keywords:** SARS-CoV-2, COVID-19, Vaccination, Modelling, Elimination, Herd immunity threshold, Vaccine effectiveness

## Abstract

**Background:** COVID-19 elimination measures, including border closures have been applied in New Zealand. We have modelled the potential effect of vaccination programmes for opening borders.

**Methods:** We used a deterministic age-stratified Susceptible, Exposed, Infectious, Recovered (SEIR) model. We minimised spread by varying the age-stratified vaccine allocation to find the minimum herd immunity requirements (the effective reproduction number R_eff_<1 with closed borders) under various vaccine effectiveness (VE) scenarios and R_0_ values. We ran two-year open-border simulations for two vaccine strategies: minimising R_eff_ and targeting high-risk groups.

**Findings:** Targeting of high-risk groups will result in lower hospitalisations and deaths in most scenarios. Reaching the herd immunity threshold (HIT) with a vaccine of 90% VE against disease and 80% VE against infection requires at least 86•5% total population uptake for R_0_=4•5 (with high vaccination coverage for 30–49-year-olds) and 98•1% uptake for R_0_=6. In a two-year open-border scenario with 10 overseas cases daily and 90% total population vaccine uptake (including 0–15 year olds) with the same vaccine, the strategy of targeting high-risk groups is close to achieving HIT, with an estimated 11,400 total hospitalisations (peak 324 active and 36 new daily cases in hospitals), and 1,030 total deaths.

**Interpretation:** Targeting high-risk groups for vaccination will result in fewer hospitalisations and deaths with open borders compared to targeting reduced transmission. With a highly effective vaccine and a high total uptake, opening borders will result in increasing cases, hospitalisations, and deaths. Other public health and social measures will still be required as part of an effective pandemic response.

**Funding:** This project was funded by the Health Research Council [20/1018].

**Research in context**

## Evidence before this study

We searched PubMed, medRxiv and SSRN for modelling studies using the term “COVID-19 vaccine AND model AND New Zealand”. We found one study by Bubar et al. which investigated age-related vaccine allocations to minimise the total deaths for countries without community transmission where total vaccination supply was limited to 50% of the population and found that direct vaccination of adults aged over 60 years nearly always reduced mortality. Moore et al. predicted a reproduction number of 1•58 after implementing vaccination in the UK and highlighted the risks of early relaxation of non-pharmaceutical interventions. Sandmann et al. also considered, in a 10-year simulation, the economic impact in the UK and suggested that with COVID-19 vaccination, small outbreaks could continue.

## Added value of this study

To our knowledge, this is the first detailed COVID-19 vaccination programme modelling for Aotearoa New Zealand, a country with closed borders and a COVID-19 elimination strategy. We forecast the effect of strategies of minimising disease spread in the community and prioritisation of high-risk age groups. We modelled different vaccination programme strategies for the following health outcomes: number of cases, hospitalisations, and deaths over two years with open borders.

## Implications of all the available evidence

To achieve the herd immunity threshold (HIT) (where R_0_=4•5), and limit community transmission (e.g. sporadic outbreaks) once borders are opened, a vaccine that has a vaccine effectiveness of 90% for disease prevention and 80% for infection reduction will require high vaccination coverage for 30–49-year-olds, and at least 86•5% total population uptake. A number of possible scenarios were modelled including where 10 overseas cases are introduced daily with open-borders and 90% total population vaccine uptake with a vaccine with VE of 90% for disease prevention and 80% for infection reduction, and prioritisation of high-risk groups for vaccination. In the two-year simulation, this scenario was forecasted to have 11,400 total hospitalisations (peak 324 active and 36 new daily cases in hospitals), and 1,030 total deaths. Where 0–11 year olds are not vaccinated and total population uptake is 80% (the maximum uptake is 84•9% and HIT is not achieved) there is an estimated 37,700 total hospitalisations (peak 2,980 active and 343 new daily cases in hospitals), and 3,120 total deaths. Other non-pharmaceutical interventions will still be required to sustain the pandemic response. These findings can support policy makers in New Zealand (including the Ministry of Health) to inform their vaccination programme and is generalisable to other countries with closed borders and elimination strategies to ensure optimal vaccination programmes.

## Introduction

COVID-19 has caused widespread morbidity and more than 4•0 million deaths globally as of July 9^th^, 2021^1^ with extensive social and economic consequences.^2^ To prevent COVID-19 outbreaks, New Zealand (NZ) adopted an early elimination strategy with non-pharmaceutical interventions, referred to as public health and social measures (PHSMs) in this paper.^3^^,^^4^

PHSMs, such as border controls, lockdown measures, quarantine, and comprehensive testing, surveillance, and contact tracing, have led to the elimination of COVID-19 transmission in NZ, but there are expectations that NZ will begin to reopen its border once the vaccination programme has progressed. Opening borders without strict isolation will continuously introduce COVID-19 to the community. The NZ government is undertaking a vaccination programme^5^ to protect NZ communities. Vaccination modelling can help anticipate potential public health outcomes based on different vaccine effectiveness (VE) reported in clinical trials^6^ and ‘real-world’ studies,[7], [8], [9], [10] and vaccination programme strategies.^5^ Estimates of the minimal vaccine coverage for herd-immunity with vaccines of different effectiveness, for instance, is needed. Vaccine allocation strategies should also take into account the potential ranges of VE in disease prevention (70–95%) and infection reduction (30–90%) from the first available vaccines including BNT162b2, mRNA-1273, and ChAdOx1 (AZD1222) vaccines.[6], [7], [8], [9], [10], [11], [12], [13]

The aim of this study was therefore to provide age-related optimisation and simulation results that can be used to design optimal vaccine programmes; including: i. achievement of HIT and, ii. if borders are open and cases of COVID-19 are introduced to the NZ community, minimisation of COVID-19 cases, hospitalisations and deaths. These include strategies to ensure maximum protection for Māori and Pasifika populations, who are at higher risk for hospitalisation and death from COVID-19.^14^^,^^15^

## Methods

We extended an age-stratified Susceptible, Exposed, Infectious, Recovered (SEIR) model^16^ with a presymptomatic phase to include vaccinated compartments (Supplemental Figure S1). The whole population is divided into eight 10-year age groups G={0–9,10–19,20–29…,60–69,70+}.

We assume that a vaccine has three effects: *e_i_* is the reduction of infection in vaccinated people (i.e. susceptibility to infection), *e_d_* is the VE for disease prevention (the default concept of VE and commonly used clinical endpoint in vaccine efficacy trials), and the third effect is reduction of infectiousness. The vaccine effect on infection reduces the susceptibility of vaccinated people by a factor *e_i_* compared with unvaccinated people. Thus, if the susceptibility of an unimmunised person in an age group *i* is *u_i_*, the susceptibility of a vaccinated person in the same age group is expected to be $u_{i}^{v}=u_{i}\left(1-e_{i}\right)$. *e_i_* has a direct influence on the viral transmission. Likewise, the probability of developing clinical disease in vaccinated infected cases in age group *i* is $\rho_{i}^{v}=\rho_{i}\left(1-e_{d}\right)/\left(1-e_{i}\right)$, where *ρ_i_* is the probability of having clinical disease in unvaccinated infected cases. *e_d_* is, thus, the effect of the vaccine on preventing disease in vaccinated individuals and corresponds to the reported vaccine efficacy and effectiveness.[6], [7], [8], [9], [10], [11], [12], [13] The effect of the vaccine on the reduction of infectiousness reduces the probability of spreading SARS-CoV-2 in vaccinated individuals. A detailed description of the model can be found in the Supplementary Appendix S1.

In addition to *e_i_*, another effect of vaccines that contributes to the change of the effective reproduction number R_eff_ is the reduction of infectiousness in vaccinated infections.^17^ This parameter is dependent on the reduction of viral shedding and/or symptoms (e.g., coughing and sneezing). In our model, it is considered that the reduction of infectiousness is a result of the reduction of clinical disease in vaccinated infections and the parameter *f* (Supplemental Table S1). This dependency is different from considering a constant reduction of infectiousness across all age groups, where different rates of symptom reduction does not influence the reduction of infectiousness in vaccinated infections. This model enables us to model the effect of *e_d_* on the overall transmission (R_eff_) while analysing the vaccine effect on reducing infection (*e_i_*).

## Model assumptions

Model assumptions included: i. For open-border modelling the behaviour of New Zealanders is as observed prior to Alert level 1 (without PHSMs). The average duration from illness onset to isolation without any intervention is 7•2 days;^3^ ii. age group sizes are constant in the open-border modelling; iii. infected, vaccinated people, without disease, have the same spreading capability as the infected asymptomatic/paucisymptomatic cases without vaccination; iv. the effectively immunised people, against either infection or disease, stay immunised with the same protection effect for the whole simulation period if they do not get re-infected. This can be interpreted as the waning vaccination effect (in the vaccinated group) being balanced by the reinforcement of the vaccination process during the simulation period. This assumption is to separate other effects from the vaccine distribution; v. vaccines are as effective for children and teenagers (age below 16) as they are for other tested age groups; vi. Māori and Pasifika populations have the same contact matrix as the whole of NZ.^18^ This assumption is, however, likely to underestimate the actual contact frequencies in this population^19^ as Māori and Pasifika people live in larger households, have larger social networks (inter-dependent households, family, church etc), have a higher proportion of the population that are young, as well as a greater likelihood of being in high exposure risk occupations;^20^ and vii. death rates (total rate and age-specific rates) are unchanged even when the active COVID-19 hospitalisations exceeds available NZ hospital capacity.^21^

## Data

We used COVID-19 case data reported in EpiSurv^22^ from February 26^th^ 2020 (when the first case was reported) to October 21^st^, 2020. COVID-19 hospitalisation rates for all age groups were inferred from recorded hospitalised cases in the national notifiable disease surveillance system, EpiSurv.^22^ We assumed that Māori and Pasifika populations have twice the hospitalisation rates estimated from EpiSurv based on previous evidence.^15^ We used the estimated age-stratified infection fatality rates modelled by Verity et al.^23^ as the age-stratified death rates for the whole of NZ, and the rates modelled by Steyn et al.^14^ as the age-stratified death rates for Māori and Pasifika populations. We used the age distribution of imported cases as recorded in EpiSurv^22^ as the age distribution of imported cases in the model (70.6% were aged 20-59 years). The susceptibility and clinical rates of COVID-19 for different ages were calculated using data from an age-stratified model published by Davies et al.^16^ A list of parameters with their source is shown in Supplemental Table S1.

## Strategies and scenarios

### Vaccine effectiveness

We investigated vaccine scenarios that only one vaccine is used for the whole population regarding the NZ vaccine plan.^5^ We analysed varying effects of the vaccine by introducing a parameter for the effectiveness on disease prevention, *e*_*d*_, and a parameter for the effectiveness on infection reduction, *e_i_*. We looked at minimum vaccine effectiveness with different uptake levels (from 60% to 100% coverage of total population) required to achieve HIT (R_eff_<1) given the R_0_ values of 2•5, 4•5, and 6.

We modelled VE (of disease prevention) in the range of 70–95%. VE of infection reduction is normally smaller than VE of disease prevention. Thus, the range of VE for infection reduction was 30% to 90% and was no greater than VE of disease prevention in all scenarios. Hereinafter, the effects of a vaccine with VE of disease prevention (*e_d_*) and VE of infection reduction (*e_i_*) is shortened to *e_d_*/*e_i_*% effectiveness for convenience. For instance, a vaccine with 95/70% effectiveness has 95% effectiveness for disease prevention and 70% effectiveness for infection reduction. The effectiveness of a vaccine is considered “uniform” when their effectiveness is equal across age groups, while the effectiveness is called “varied” when the vaccine effectiveness is reduced in older age groups. The current vaccination strategy in NZ focuses on two dose vaccination, rather than maximising the number of administrations of first dose. The second dose is administered at least 21 days after the first dose.^5^

### Vaccine strategies with closed borders

In this study, we compared two vaccine strategies, where each could be implemented through one of the following optimisation criteria: (1) minimising the effective reproduction number or the spreading rate; and (2) minimising disease in the total high-risk population (risk for severe disease and deaths). The first strategy minimises the leading eigenvalue of the next generation matrix (i.e. R_eff_) or the spreading rate. This strategy requires minimum requirements for vaccine effectiveness and the total uptake to achieve HIT. Therefore, it is used to analyse the minimum herd immunity requirements. The total high-risk population in the second strategy can be estimated as $\sum_{i}S_{i}d_{i}$, which are the age-stratified susceptible populations (*S_i_*) weighted by their mortality rates due to COVID-19 (*d_i_*). This strategy begins with vaccination in the oldest groups, followed by the younger groups, because older groups are known to have higher risks for both severe disease and death.^24^^,^^25^ Hereafter, two strategies are referred to as the spread-minimising/minimise R_eff_ strategy and the high-risk (group) targeting strategy respectively. A third strategy that balances between these two strategies is included in Supplemental Appendix S2.

**Figure 1:**
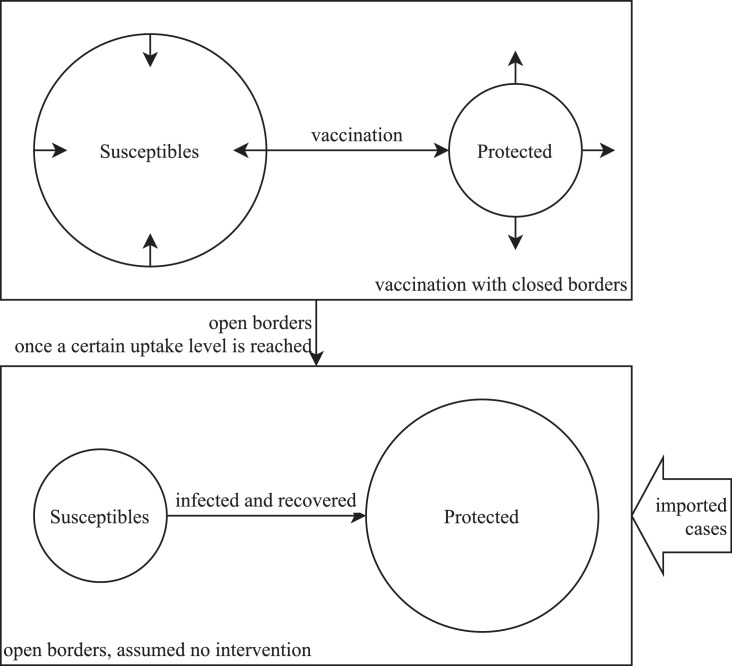
Pre-transmission vaccination process. Note: A level of uptake (60–100% total population) has been reached before opening borders.

Both strategies are assumed to be implemented with closed borders until a certain uptake level is reached, i.e. from 60 to 100% total population coverage (Figure 1). A vaccination uptake of 80–90% of the NZ population requires vaccinating individuals aged under 16 and a higher rate of vaccination than being achieved in other countries. In the United Kingdom, Israel, and Canada,^26^ around 60% of total populations have been vaccinated with more than 95% in older age groups.

We assumed the following constraints on all vaccine strategies: i. each age group is vaccinated at least 20%, except for the 70+ year olds with minimum 80% vaccine coverage. ii. the maximum coverage for each age group is 90% for variants with lower R_0_ values (2–3•5) and 100% for variants with higher R_0_ values (4•5–6). The range of the higher R_0_ values corresponds to the early estimates of R_0_ values for the variants of concern (4•5–6).

To compare these strategies, we ran two-year simulations of two vaccine strategies with open borders, where a continuous vaccination process is assumed to mitigate any potential waning effect of the vaccine (Figure 1). We assumed there is a constant ten daily imported cases that become part of the community, which are equivalent to a total of 7,300 imported cases. As part of a sensitivity analysis, we also modelled on 100 daily imported cases (73,000 total). Imported cases are assumed to be unvaccinated. Comparison criteria include total COVID-19 deaths, total community cases, peak active cases, total hospitalisations, and peak active hospitalised cases (peak hospitalisations). The measures relating to hospitalisations and deaths include a predicted 444 total hospitalised and 84 deaths from 7,300 imported cases (Supplemental Appendix S2). As vaccination has not been approved for 0–15 year olds in New Zealand,^5^ we carried out a sensitivity analysis where uptake was 0% for 0–9 year olds and the vaccine coverage of 10–19 year olds is assumed to have a maximum level of 35% as the subgroup of 16–19 year olds contribute nearly 40% to the group of 10–19 year olds.^27^ We also limited our analysis to 0–11 year olds (as clinical trials have yet to release findings). For this analysis, the vaccine coverage of 10–19 year olds is therefore assumed to have a maximum level of 70% as the subgroup of 12–19 year olds contribute about 79% to the group of 10–19 year olds.

## Ethics and permissions

The study protocol was approved by the Health and Disability Ethics Committee, New Zealand, under the protocol number 20/NTB/156.

## Role of the funding source

The sponsors of the study had no role in study design, data collection, data analysis, data interpretation, or the writing of this report.

## Results

### Minimum herd immunity requirements

**Figure 2:**
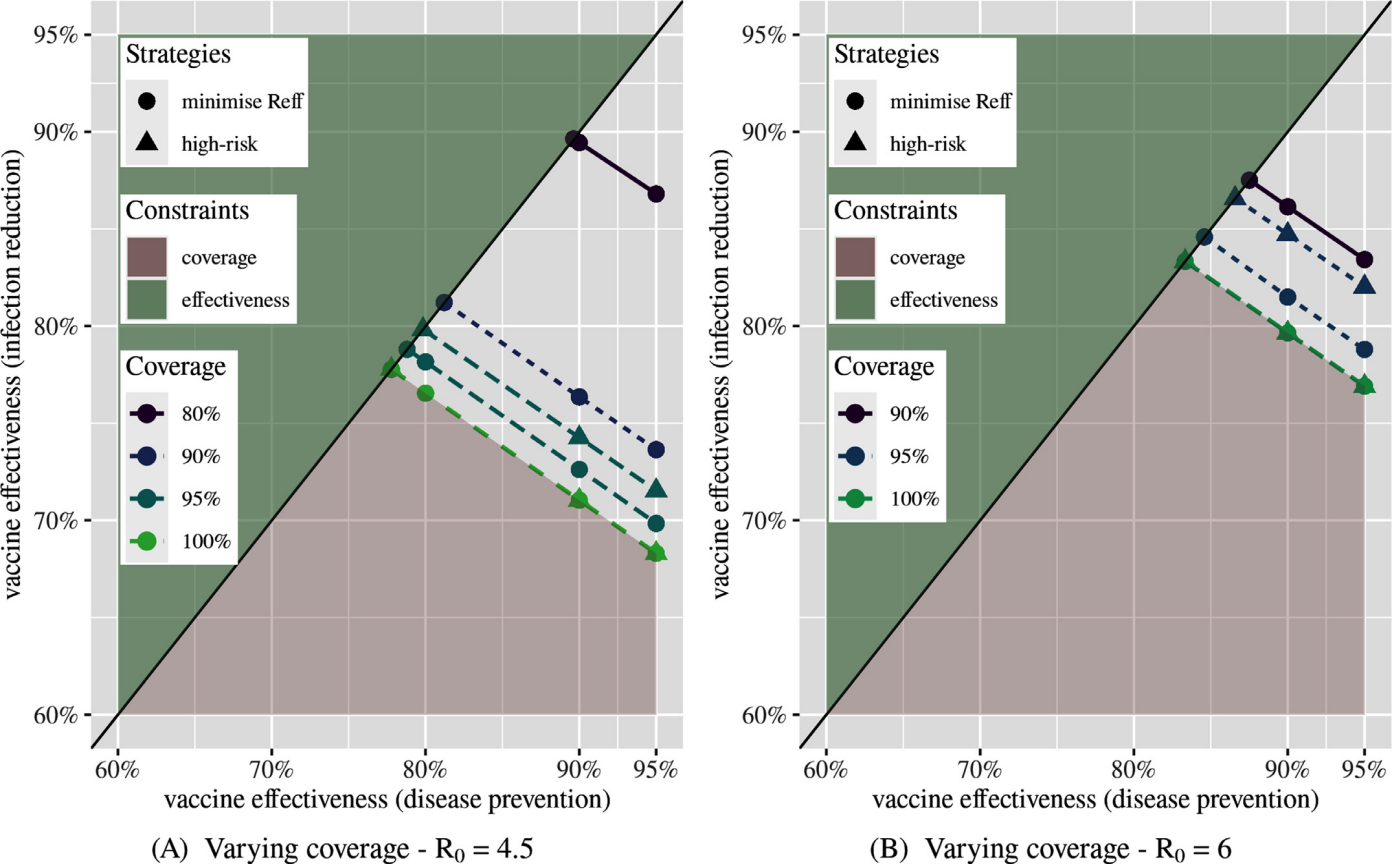
Vaccine effectiveness and New Zealand population vaccine uptake requirements for herd immunity threshold. Note: The minimal vaccine effectiveness on infection reduction and disease prevention for the herd immunity threshold at multiple vaccine uptake levels: (A) R_0_=4•5 and (B) R_0_=6. The spread-minimising strategy (i.e. minimise R_eff_) offers lower requirements of vaccine effectiveness (on both effects) than the high-risk targeting strategy given the same uptake levels. Both effects are considered equal across age groups in this analysis. As the vaccine effectiveness on infection reduction is expected to be not greater than the vaccine effectiveness on disease prevention, all herd immunity lines are limited to the bottom half of the plot (divided by the black line).

Figure 2 (A-B) and Figure 4 (A-D) show minimum herd immunity requirements for two vaccine strategies at multiple uptake levels given the R_0_ value is in the range of 4.5–6 and 2–3.5 where there is a minimum 80% vaccine uptake for high risk groups. Reaching the HIT with a vaccine of 90/80% effectiveness requires at least 86.5% total population uptake for R_0_=4.5 and 98.1% uptake for R_0_=6 with high vaccination coverage for 30–49-year-olds, i.e. the spread-minimising strategy. With the same vaccine and the high-risk targeting strategy, reaching HIT requires 92% and 99.2% total population uptake levels for R_0_=4.5 and 6 respectively. With 90% total population coverage with a vaccine of 90% VE for disease prevention, a minimum 76% VE of infection reduction for R_0_=4.5 and 86% VE of infection reduction for R_0_=6 is required (using the spread-minimising strategy). For 80% population vaccine coverage, a VE of 87% for infection reduction is needed. For all VE scenarios (Figures 2, 4, Supplemental Appendix S3), the spread-minimising strategy has the minimum requirements of VE for HIT among vaccine strategies given the same uptake levels although it may not be optimal for protecting the whole population from the risk of hospitalisations and deaths. Vaccinating the age groups 30–39 and 40–49 can minimise the initial effective reproduction numbers (given a limited number of doses), while 60+ and 0–9 are the age groups that contribute the least to the reduction of the effective reproduction number and the achievement of HIT.

### Open border modelling results

**Figure 3:**
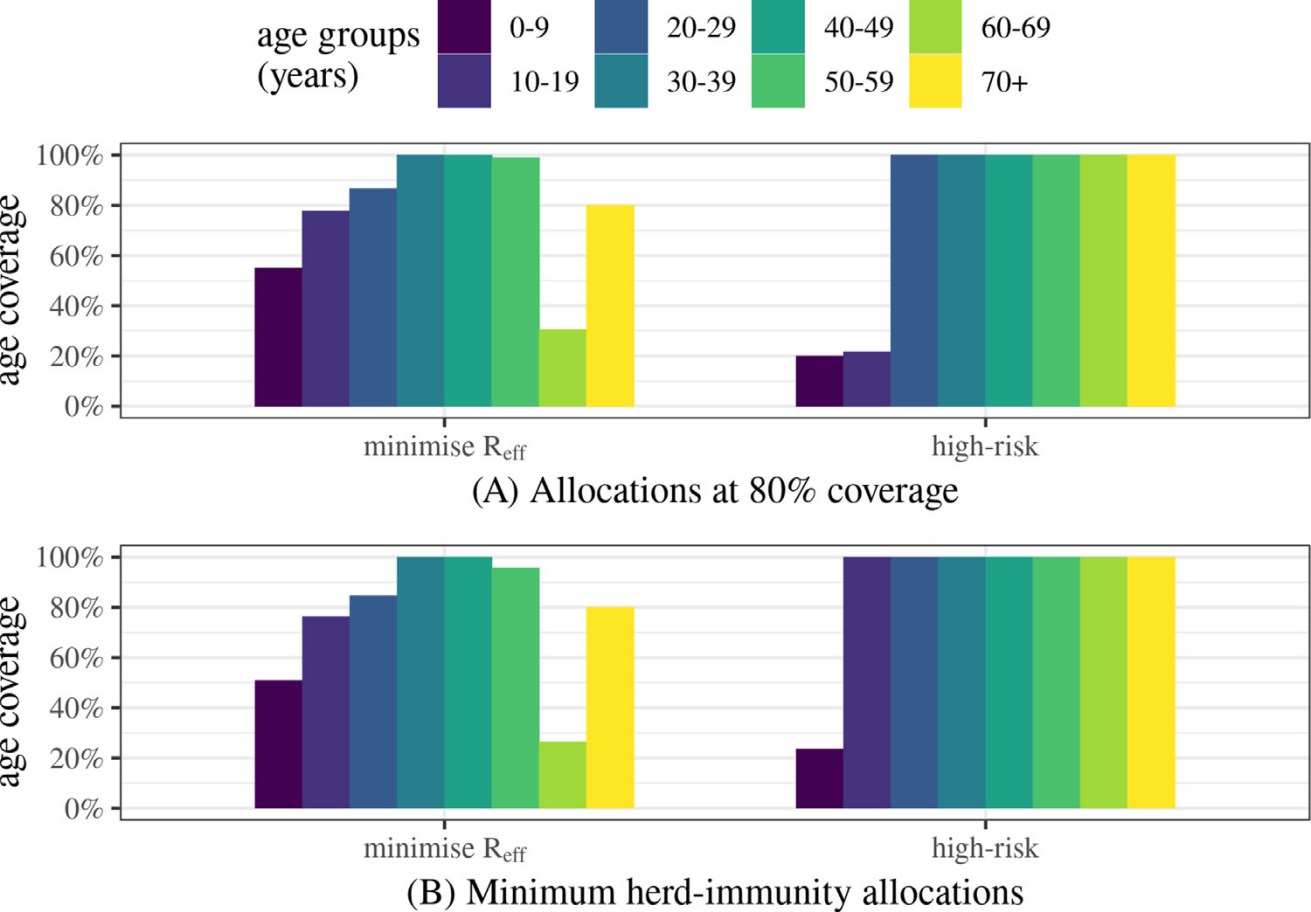
Age-stratified allocations for two strategies with a vaccine of 95/90% uniform effectiveness and 80% coverage (A), and their minimum herd-immunity allocations (R0=4•5) (B). Note: Illustration of vaccine allocations for two strategies (i.e. minimising R_eff_ and prioritising high-risk groups). A – shows 80% coverage with 95% (uniform) effectiveness on disease prevention and 90% (uniform) of infection reduction. B – shows the minimal age-stratified allocations required for HIT by the corresponding strategies. The high-risk targeting strategy requires more than 80% coverage (∼90•5%) to achieve HIT, while the spread-minimising strategy needs less vaccine uptake for HIT (78•2% total coverage). For R_0_=6.0 near complete coverage for all age groups is required to achieve the herd-immunity threshold.

The differences in vaccine allocation of the investigated strategies can be found in Figure 3. The spread-minimising strategy (minimise R_eff_) in this figure has enabled HIT at 80% total population coverage. Probable scenarios of VE and vaccine uptake levels in a two-year simulation of the model can be found in Table 1 (open borders, ten cases daily introduced to the community and R_0_=4.5). Further vaccine scenarios for the whole of NZ can be found in Supplemental Tables S2–8.

**Table 1:** Comparison of cases, hospitalisations, and deaths in New Zealand population (R_0_=4•5) – 10 imported cases per day with open borders or 7,300 total imported cases with two-year open borders

Vaccine scenarios (*e_d_*/*e_i_* & uptake)	Vaccine strategies	Peak active cases	Total community cases	Peak hosps.	Total hosps.	Total deaths
95/90% uniform – 100% coverage	n/a	133	5,010	7	587	64
95/90% uniform	minimise R_eff_	217	12,100	15	1,170	173
90% coverage	high-risk	1,380	80,400	34	1,950	145
	hybrid	270	16,800	10	814	77
95/90% uniform	minimise R_eff_	1,090	75,700	78	5,210	863
80% coverage	high-risk	66,700	821,000	1,530	17,400	947
	hybrid	1,950	127,000	123	7,490	1,150
95/90% uniform	minimise R_eff_	46,500	974,000	2,910	55,200	8,140
70% coverage	high-risk	163,000	1,500,000	3,640	31,700	1,710
95/80% uniform – 100% coverage	n/a	272	18,100	9	708	76
95/80% uniform	minimise R_eff_	584	43,500	29	2,120	333
90% coverage	high-risk	7,050	318,000	127	5,130	383
	hybrid	803	60,100	28	2,010	261
95/80% uniform	minimise R_eff_	16,600	673,000	908	32,200	5,440
80% coverage	high-risk	110,000	1,360,000	2,050	22,800	1,400
95/80% uniform	minimise R_eff_	122,000	1,790,000	5,840	75,400	11,100
70% coverage	high-risk	240,000	2,140,000	4,480	36,900	2,190
95/70% uniform – 100% coverage	n/a	1,560	119,000	24	1,630	168
95/70% uniform	minimise R_eff_	14,600	734,000	459	19,600	3,210
90% coverage	high-risk	42,600	1,180,000	575	13,700	1,130
95/70% uniform	minimise R_eff_	91,900	1,820,000	3,560	60,100	9,910
80% coverage	high-risk	188,000	2,200,000	2,800	28,900	1,960
95/70% uniform	minimise R_eff_	236,000	2,650,000	8,970	87,900	12,900
70% coverage	high-risk	341,000	2,830,000	5,330	40,400	2,530
95/60% uniform – 100% coverage	n/a	59,500	1,590,000	561	12,700	1,330
95/60% uniform	minimise R_eff_	119,000	2,190,000	2,500	39,300	6,260
90% coverage	high-risk	146,000	2,340,000	1,540	21,200	1,870
95/60% uniform	minimise R_eff_	221,000	2,800,000	6,670	72,400	11,900
80% coverage	high-risk	303,000	2,990,000	3,670	32,300	2,300
95/60% uniform	minimise R_eff_	370,000	3,330,000	11,700	92,600	13,700
70% coverage	high-risk	455,000	3,390,000	6,060	41,600	2,670
90/80% uniform – 100% coverage	n/a	337	23,200	15	1,140	120
90/80% uniform	minimise R_eff_	918	67,500	54	3,750	559
90% coverage	high-risk	11,100	439,000	324	11,400	1,030
	hybrid	1,470	105,000	71	4,640	609
90/80% uniform	minimise R_eff_	29,200	928,000	1,780	50,100	7,890
80% coverage	high-risk	122,000	1,500,000	3,430	38,800	3,170
90/80% uniform	minimise R_eff_	145,000	1,960,000	7,770	94,400	13,200
70% coverage	high-risk	259,000	2,260,000	7,020	58,600	4,590
90/70% uniform – 100% coverage	n/a	5,170	329,000	130	7,060	720
90/70% uniform	minimise R_eff_	34,000	1,200,000	1,310	39,700	5,810
90% coverage	high-risk	63,100	1,490,000	1,480	30,100	2,880
90/70% uniform	minimise R_eff_	121,000	2,080,000	5,400	80,600	12,200
80% coverage	high-risk	212,000	2,390,000	4,910	49,900	4,320
90/70% uniform	minimise R_eff_	267,000	2,810,000	11,400	107,000	15,000
70% coverage	high-risk	366,000	2,950,000	8,400	63,800	5,220
90/60% uniform – 100% coverage	n/a	93,700	1,940,000	1,740	30,600	3,260
90/60% uniform	minimise R_eff_	157,000	2,460,000	4,430	59,900	8,590
90% coverage	high-risk	183,000	2,590,000	3,400	41,700	4,150
90/60% uniform	minimise R_eff_	262,000	3,000,000	8,900	89,400	13,300
80% coverage	high-risk	335,000	3,140,000	6,420	54,400	4,860
90/60% uniform	minimise R_eff_	404,000	3,440,000	14,700	113,000	15,800
70% coverage	high-risk	483,000	3,490,000	9,460	64,400	5,380
80/70% uniform – 100% coverage	n/a	41,800	1,310,000	2,000	55,000	5,780
80/70% uniform	minimise R_eff_	95,100	1,930,000	5,170	93,500	11,900
90% coverage	high-risk	122,000	2,090,000	5,340	81,600	8,510
80/70% uniform	minimise R_eff_	190,000	2,560,000	10,800	131,000	17,500
80% coverage	high-risk	268,000	2,770,000	11,000	105,000	10,600
80/70% uniform	minimise R_eff_	333,000	3,100,000	18,000	156,000	20,000
70% coverage	high-risk	419,000	3,190,000	16,200	119,000	11,800
80/60% uniform – 100% coverage	n/a	175,000	2,510,000	6,290	79,400	8,690
80/60% uniform	minimise R_eff_	244,000	2,930,000	9,990	107,000	13,600
90% coverage	high-risk	265,000	3,000,000	9,130	92,600	9,940
80/60% uniform	minimise R_eff_	340,000	3,300,000	15,500	138,000	18,600
80% coverage	high-risk	406,000	3,420,000	13,600	106,000	10,900
80/60% uniform	minimise R_eff_	475,000	3,650,000	21,500	155,000	20,200
70% coverage	high-risk	543,000	3,680,000	17,600	115,000	11,600
70/60% uniform – 100% coverage	n/a	266,000	2,940,000	13,900	141,000	15,800
70/60% uniform	minimise R_eff_	336,000	3,280,000	18,300	165,000	20,100
90% coverage	high-risk	354,000	3,330,000	17,600	154,000	17,100
70/60% uniform	minimise R_eff_	426,000	3,570,000	23,700	187,000	23,600
80% coverage	high-risk	483,000	3,660,000	22,900	166,000	18,100
70/50% uniform – 100% coverage	n/a	421,000	3,520,000	17,500	136,000	15,600
70/50% uniform	minimise R_eff_	491,000	3,780,000	21,400	154,000	19,000
90% coverage	high-risk	503,000	3,800,000	20,200	144,000	16,300
90/80%: age 50+70/60%: younger 80% coverage	dual vaccine	175,000	2,180,000	8,410	95,300	12,000

Peak active and total community cases do not include imported cases. All measures related to hospitalisations and deaths (in all scenarios) include imported cases, which are equivalent to the expectations of 444 total hospitalisations and 49•6 total deaths.

Note: Forecasts for a two-year simulation. *e_d_* is VE of disease prevention. *e_i_* is VE of infection reduction. The total community cases include vaccinated cases, who are less likely to develop symptoms, need hospitalisation or die than unvaccinated individuals. A scenario of “95/90% uniform, 80% coverage” means that the vaccine has uniform effects across age groups with 95% disease prevention and 90% infection reduction, and the uptake is 80% coverage of total population. HIT is not achievable in the third and fourth scenarios, where the vaccine has poor effectiveness on infection reduction. The last scenario has 80% vaccine uptake when two vaccines are available. The “dual vaccines'' strategy reused the vaccine allocation from the high-risk (group) targeting strategy. This dual strategy allocated a vaccine with lower effectiveness 70/60% for the five younger age groups and the 90/80% vaccine for the three oldest groups (aged 50 and over). Targeted vaccine strategies: (minimise R_eff_) Targeting of younger (socialised) age groups to minimise R_eff_; (high-risk) Groups susceptible to hospitalisation and death. Results are rounded to the third significant number. The lowest values that are at least 10% lower than other corresponding numbers of the same scenarios are in bold.

The spread-minimising strategy (i.e. minimise R_eff_) resulted in the smallest peak and total community cases in all scenarios (assuming the vaccine can reduce infection *e_t_* > 0). The strategy which targeted high-risk groups yielded the fewest hospitalisations (active or total) and total deaths in the majority of modelled scenarios (Table 1). For the high-risk group targeting strategy, a high total vaccine uptake is required that is enough to also cover young adults to achieve better outcomes in general. For instance, in a scenario with R_0_=4.5 and a vaccine having a VE of 90/70% and 90% population uptake, the high-risk group targeting strategy was forecasted to have the lowest number of deaths and total hospitalisations, i.e. 2,880 vs 5,810 fatalities and 30,100 vs 39,700 hospitalisations (peak active hospitalisations 1,480 vs. 1,310) respectively, and more community cases than the spread-minimising strategy, i.e. a total of 1,490,000 vs. 1,200,000 cases (peak active community cases 63,100 vs. 34,000). Where the R_0_ value is 6 and 90% total population uptake with the same vaccine, modelling the high-risk group targeting strategy resulted in lower hospitalisations and deaths but higher cases than the spread-minimising strategy, i.e. 6,100 vs. 11,700 deaths, 59,600 vs. 82,600 hospitalisations (peak active 5,960 vs. 7,320), 2,860,000 vs. 2,750,000 cases (peak active community cases 253,000 vs. 213,000).

A dual vaccine approach has been investigated where the vaccine distribution follows the high-risk targeting strategy (Table 1). All groups aged 50 and over are allocated with a vaccine of 90/80% effectiveness and the rest are allocated with a vaccine of lower 70/50% effectiveness. The outcomes of this scenario are 2,180,000 cases (peak active 175,000 cases), 95,300 hospitalisations (peak active 8,410 in hospital), and total 12,000 fatalities. These numbers are in between the corresponding outcomes of two scenarios using either one of the two vaccines.

We have modelled vaccine scenarios of immunesenescence with a 50% reduction in effectiveness (for both disease prevention and infection reduction) in people aged 60 and over (Supplemental Table S9). We also analysed the sensitivity of the results on the assumed average daily imported cases and the synthetic contact matrix^18^ in Supplemental Appendix S4. Customised vaccine strategies and open-border modelling results for Māori and Pasifika populations are provided in Supplemental Appendix S5.

## Vaccination excluding youngest age-groups

Where vaccination is not allocated to the 0–15 year olds^5^ or the 0–11 year olds, the maximum attainable total population vaccine coverage is 79.8% or 84.9%. At a high R_0_ value of 4.5 or higher, these maximum total coverage levels are not enough to achieve HIT. Therefore, opening borders without vaccinating the under-12 group or the under-16 group were predicted to result in a large number of cases, hospitalisations, and deaths (Table 2 and Supplemental Table S2). For instance, where 0–11 year olds are not vaccinated and R_0_=4.5 (Table 2), the high-risk targeting strategy with a high uptake level 80% (over the maximum 84.9%) and a vaccine of 90/80% effectiveness was predicted to have lower deaths and total hospitalisations and more community cases, i.e. 3,120 vs. 5,850 deaths, 37,700 vs. 44,100 hospitalisations (peak 2,980 vs 2,630), 1,480,000 vs 1,180,000 cases (peak 107,000 vs 62,700).

**Table 2:** Comparison of cases, hospitalisations and deaths when vaccination is not allocated to the 0–11 year olds (R_0_=4•5) – 10 imported cases per day with two-year open borders

Vaccine scenarios (*e_d_*/*e_i_*, uptake, & R_0_)	Vaccine strategies	Peak active cases	Total community cases	Peak hosps.	Total hosps.	Total deaths
95/90% uniform – 84•9% coverage	n/a	17,900	413,000	388	8,310	513
95/90% uniform	minimise R_eff_	23,400	511,000	918	18,300	2,500
80% coverage	high-risk	50,600	783,000	1,140	16,100	909
95/90% uniform	minimise R_eff_	64,100	1,050,000	3,510	52,400	8,070
70% coverage	high-risk	157,000	1,490,000	3,490	31,100	1,690
95/90% uniform	minimise R_eff_	179,000	1,860,000	10,000	95,900	12,500
60% coverage	high-risk	310,000	2,180,000	7,860	53,800	2,680
95/90% uniform	minimise R_eff_	361,000	2,570,000	19,500	133,000	15,500
50% coverage	high-risk	508,000	2,790,000	15,300	84,800	3,980
95/80% uniform – 84•9% coverage	n/a	39,000	826,000	695	13,100	894
95/80% uniform	minimise R_eff_	51,000	1,010,000	1,690	29,300	4,110
80% coverage	high-risk	93,500	1,330,000	1,710	21,700	1,370
95/80% uniform	minimise R_eff_	128,000	1,780,000	5,700	70,000	10,600
70% coverage	high-risk	232,000	2,130,000	4,290	36,400	2,150
95/80% uniform	minimise R_eff_	279,000	2,560,000	13,100	108,000	14,100
60% coverage	high-risk	403,000	2,780,000	8,750	57,200	3,010
95/80% uniform	minimise R_eff_	466,000	3,150,000	22,000	139,000	16,400
50% coverage	high-risk	604,000	3,300,000	16,000	85,700	4,080
95/70% uniform – 84•9% coverage	n/a	99,200	1,730,000	1,390	21,000	1,590
95/70% uniform	minimise R_eff_	122,000	1,960,000	3,200	44,100	6,300
80% coverage	high-risk	174,000	2,180,000	2,540	28,100	1,950
95/70% uniform	minimise R_eff_	233,000	2,630,000	8,400	82,700	12,500
70% coverage	high-risk	331,000	2,830,000	5,130	40,100	2,510
95/70% uniform	minimise R_eff_	399,000	3,220,000	15,700	114,000	15,000
60% coverage	high-risk	506,000	3,320,000	9,500	58,500	3,150
95/70% uniform	minimise R_eff_	576,000	3,640,000	24,100	141,000	16,800
50% coverage	high-risk	698,000	3,720,000	16,600	85,200	4,030
90/80% uniform – 84•9% coverage	n/a	47,900	973,000	1,360	24,700	2,140
90/80% uniform	minimise R_eff_	62,700	1,180,000	2,630	44,100	5,850
80% coverage	high-risk	107,000	1,480,000	2,980	37,700	3,120
90/80% uniform	minimise R_eff_	148,000	1,950,000	7,550	89,200	12,800
70% coverage	high-risk	250,000	2,250,000	6,750	58,000	4,580
90/80% uniform	minimise R_eff_	303,000	2,680,000	15,700	128,000	16,200
60% coverage	high-risk	422,000	2,860,000	12,200	81,700	5,930
90/70% uniform - 84•9% coverage	n/a	123,000	1,980,000	2,860	40,300	3,690
90/70% uniform	minimise R_eff_	148,000	2,190,000	5,020	64,900	8,660
80% coverage	high-risk	199,000	2,380,000	4,580	49,200	4,310
90/70% uniform	minimise R_eff_	262,000	2,800,000	10,900	104,000	14,700
70% coverage	high-risk	356,000	2,960,000	8,140	63,500	5,220
90/70% uniform	minimise R_eff_	426,000	3,320,000	18,700	134,000	17,100
60% coverage	high-risk	529,000	3,400,000	13,200	82,700	6,130
80/70% uniform – 84•9% coverage	n/a	184,000	2,460,000	7,790	93,900	9,670
80/70% uniform	minimise R_eff_	213,000	2,640,000	10,500	118,000	14,600
80% coverage	high-risk	258,000	2,770,000	10,600	104,000	10,600
80/70% uniform	minimise R_eff_	327,000	3,100,000	17,300	152,000	19,700
70% coverage	high-risk	411,000	3,210,000	15,900	120,000	11,800
80/70% uniform	minimise R_eff_	483,000	3,510,000	25,600	178,000	21,800
60% coverage	high-risk	576,000	3,560,000	22,000	137,000	13,000
80/60% uniform – 84•9% coverage	n/a	328,000	3,240,000	11,100	99,500	10,500
80/60% uniform	minimise R_eff_	358,000	3,360,000	14,400	124,000	15,500
80% coverage	high-risk	398,000	3,430,000	13,200	106,000	11,000
80/60% uniform	minimise R_eff_	468,000	3,660,000	20,700	152,000	20,100
70% coverage	high-risk	537,000	3,710,000	17,400	116,000	11,600
80/60% uniform	minimise R_eff_	610,000	3,920,000	27,900	173,000	21,600
60% coverage	high-risk	683,000	3,930,000	22,500	130,000	12,300
70/50% uniform – 84•9% coverage	n/a	555,000	3,920,000	21,900	148,000	16,700
70/50% uniform	minimise R_eff_	671,000	4,160,000	31,200	189,000	24,300
70% coverage	high-risk	717,000	4,160,000	27,500	159,000	17,400

Note: NZ's vaccination plan has not included vaccinating 0–11 year olds.^5^ The total population coverage is therefore no more than 84•9% (other age groups have a maximum coverage of 100%).^27^ At the maximum total coverage (84•9%), both vaccine strategies become identical.

**Figure 4:**
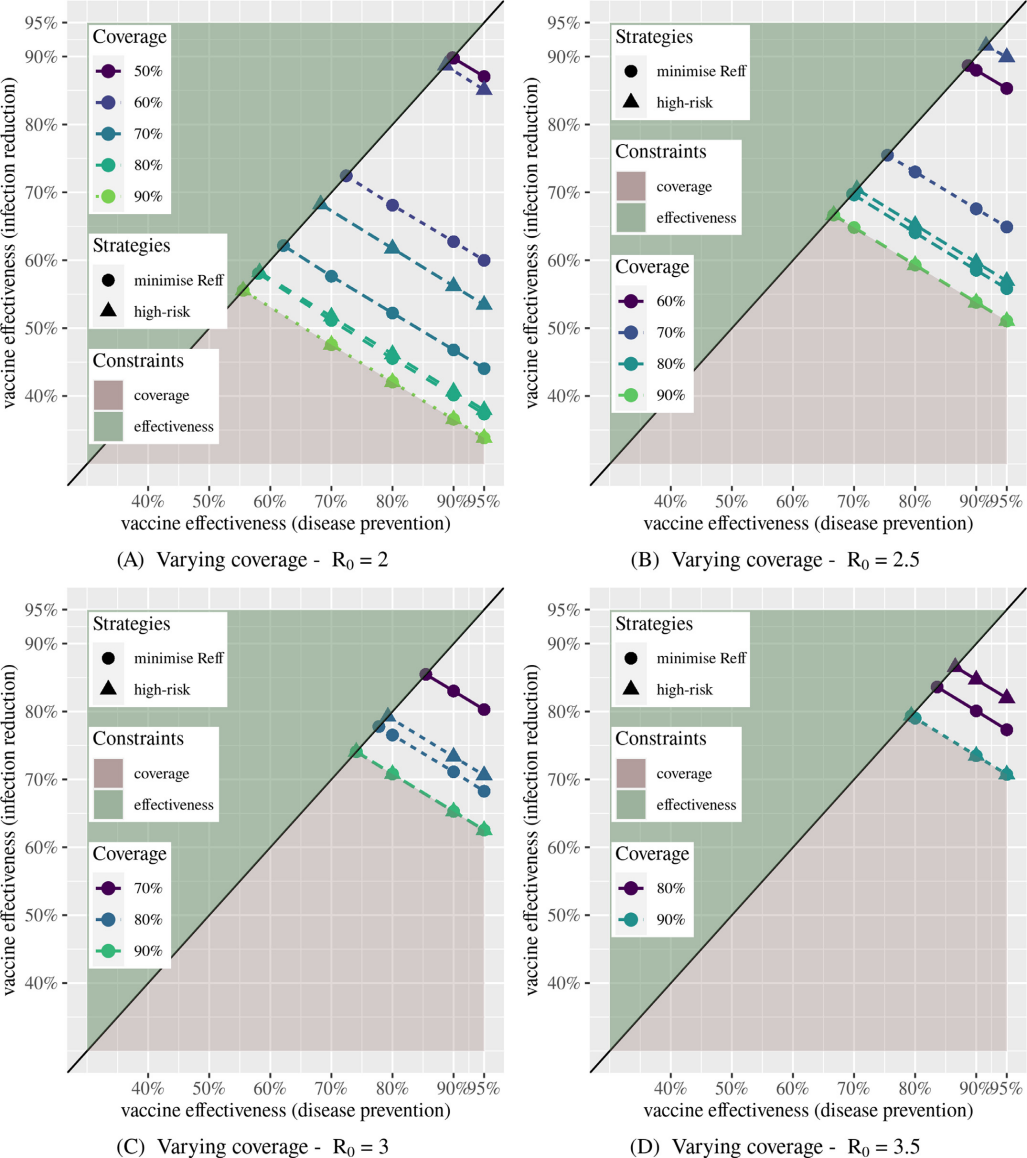
Vaccine effectiveness and New Zealand population vaccine uptake requirements for the herd immunity threshold at lower R0 values. Note: The minimal vaccine effectiveness on infection reduction and disease prevention for HIT at multiple vaccine uptake levels: (A) R_0_=2; (B) R_0_=2•5; (C) R_0_=3; and (D) R_0_=3•5. The maximum vaccine coverage in each age group is 90%.

At a lower R_0_ value of 2.5 (Figure 4 and Supplemental Figures S5-6), the achievement of HIT will require a minimum VE against infection of 61% for excluding 0–15 year olds and 73% for excluding 0–11 year olds with the limits of 76.4% and 71.8% respectively (maximum 90% coverage for each age group). The open border modelling outcomes have higher numbers of cases, hospitalisations, and deaths in almost all scenarios and vaccine strategies compared with vaccinating all age groups.

## Discussion

Reaching HIT will prevent widespread community outbreaks and, as a result, vulnerable populations will have a greater chance of protection from severe disease. A long-term lockdown may only postpone future outbreaks if a high level of immunity (by vaccination or natural immunity) is not targeted. Achieving HIT through vaccination in New Zealand while borders are closed will require an effective vaccine that can reduce infection, and high national vaccine uptake. Achievement of HIT without vaccinating the youngest age groups will require a vaccine with higher VE against infection. In an open border scenario with the relaxation of PHSMs and a highly effective vaccine for both disease prevention and infection reduction, targeting high-risk groups (including Māori and Pasifika) and achieving a high national uptake level, e.g. 80%, will result in a relatively low number of forecasted COVID-19 hospitalisations and deaths by international comparisons.^28^ Where the vaccine has lower VE for infection reduction, more COVID-19 cases, hospitalisations and deaths are likely.

A strategy to achieve HIT will ensure limited community transmission (e.g. sporadic outbreaks) once borders are opened but would require a vaccine with a minimum 87% VE for infection reduction (where R_0_=4.5) and a high vaccine coverage rate of 80% total population. This estimated VE for infection reduction is higher than the 85% effectiveness for preventing infections that was predicted to result in a reproduction number of 1.58 in the UK. This study did not however account for further reduced viral shedding from vaccinated individuals, reducing onward transmission.^28^

Although, HIT is potentially possible e.g. with recent evidence of the BNT162b2 vaccine’s effect against infection,^29^ it is also possible that emerging effectiveness challenges against new virus variants will necessitate a shift in focus away from herd immunity strategies to protection of at-risk individuals against severe disease.^30^ Although the range of estimated VE used in this study are plausible, in particular for the mRNA vaccines licensed in NZ,^11^ the lower bounds of VE may need to be extended in the presence of variants of concern.^31^

Comparisons of our forecast peaks (with 80% uptake, and 95% VE for disease and 70% for infection) with other countries who had widespread community transmission during the first waves of disease (with no available vaccination) can be made. Scotland has a broadly comparable population size but higher population density (e.g. Scotland, UK, 5.4m vs. 5.1m population, 19.0/km^2^ vs. 67.2/km^2^). Variants of concern with high R_0_ values such as Alpha ^32^ and Delta variants,^33^ were dominant in Scotland in Spring 2021. In an open border scenario, our NZ model for R_0_=4.5, where a vaccine of 90/80% effectiveness is not allowed for individuals aged under 16, has estimated a peak of 355 new daily hospitalisations (3,090 peak active hospitalised cases) vs. 92 peak daily hospitalisations found during the ongoing wave in Scotland (from June until July 2021), and a higher peak of daily cases 14,800 (including asymptomatic cases, 110,000 peak active cases) vs. 3,930 found in Scotland with 64.7% two-dose vaccine coverage and 88.1% first-dose vaccine coverage of all people aged 18 and over.^34^ The numbers hospitalisations and deaths for NZ will be higher as this includes 7,300 unvaccinated imported cases.

Several studies have addressed COVID-19 vaccination strategies. Bubar et al.^35^ compared five vaccine strategies that allocate vaccine doses on ‘under 20’, ‘adults 20–49’, ‘adults 20+’, ‘adults 60+’, and ‘all ages’ in terms of the reduction of deaths and infections. This study focused on the initial phase of vaccination, modelling a total vaccine uptake of no more than 50% of the population and applying non-pharmaceutical interventions to reduce the spreading rate. Moore et al. predicted 96,700 deaths (51,800–173,200) if interventions are removed after vaccination with a vaccine that could prevent 85% infections.^28^ Sandmann et al. used an age-structured transmission and economic model to estimate the economic impact of vaccination for the UK in a ten-year simulation.^36^ This study suggested that vaccination could add substantial health and economic value and population-wide physical distancing might not be justifiable.

Compared with other models used for vaccination studies, the SEIR model used in this study provides a model with fewer assumptions for the same disease dynamics. By grouping individuals of the same disease phase into a compartment, this SEIR model approach only requires transitions among phases instead of requiring numerous rules representing all the disease phases that are used in agent-based models. Although agent-based models have been used to apply a number of assumptions which are useful for understanding the effect of multiple public health interventions, they have limitations due to being computationally demanding.^37^ For instance, agent-based models do not integrate age groups, but use averages for the whole population, whereas we know that vaccine distribution across age groups is unlikely to be uniform.^38^ The required uptake levels for HIT are subject to an estimated basic reproduction number R_0_ of COVID-19 in NZ, national priorities and consideration to protect health and social care workers and the most clinically susceptible groups. While the R_0_ value for NZ has not been reliably estimated, its actual value is also probably dependent on seasonality.^39^^,^^40^ Moreover, R_0_ is likely to increase with the emergence of the new virus strains.^41^^,^^42^ To consider possible increases in R_0_, a strength of this study is that we also investigated herd immunity requirements for higher R_0_ values (4.5 and 6). These R_0_ values could be the potential reproduction number of new variants. However, this study does not include changing R_0_ values over time (with the introduction of new variants of concern). Rather R_0_ values are fixed for the two-year period. Another strength of this work is that the model can be calibrated when more accurate parameter values are available. There is uncertainty with new variants of concern. Model parameters, such as R_0_, latent/infectious periods, and age-structured mortality rates may therefore vary. However, further parameters can be added to the model once evidence of new parameters emerges.

The safe opening of borders in NZ will be dependent on a vaccine that has high effectiveness against both COVID-19 disease and viral transmission. A limitation of our study is that there is still some uncertainty regarding the vaccine effectiveness against transmission. Therefore, modelling strategies and scenarios and forecasting their potential impact on the NZ population with more accurate assumptions (including infection reduction and waning vaccine immunity) needs further investigation. A further limitation is uncertainty around the potential number of imported cases in particular if travel is restricted from regions with high numbers of cases. There is also uncertainty regarding immunesenescence and our assumption of uniform effectiveness across age groups may not hold, although we have modelled vaccine scenarios with a reduction in effectiveness (for both disease prevention and infection reduction) in people aged 60 and over. The targeting of high-risk groups (in an open border scenario), in this case, may not yield the lower total deaths in many scenarios as the disease prevention effect is now lower. This is in contrast to another modelling study which found that, in the event of low effectiveness amongst older adults and no more than 50% uptake level, the advantage of prioritising all adults or adults 20–49 vs. adults 60+ was small.^35^

This work provides data on a range of vaccine scenarios and strategies to inform NZ vaccine planning.^5^ While research to estimate vaccine effectiveness for reducing severe outcomes and infection is underway, a 70% VE against infection is predicted to be the minimum required to achieve HIT for NZ with an R_0_=4.5 and 95% total vaccine coverage. As NZ's vaccination plan has not yet included those aged 0–15 years for vaccination,^5^ achievement of HIT without vaccinating this group may be impossible, especially if the imported cases are Alpha or Delta variants of concern.^33^ Thus, to help reduce cases, hospitalisations, and deaths, other public health interventions will be required to manage the public health response.

## Contributors

CRS, MA, BN, JdL, JG, SJ, MB, WS, AS, NF, DM and DW conceived this study. All commented on the paper, oversaw the analysis and edited the final manuscript. TN, MA, BN and CS led the writing of the paper. TN, RD, MA cleaned and analysed the data. All authors contributed to the study design. All authors contributed to drafting the paper and revised the manuscript for important intellectual content. All authors gave final approval of the version to be published.

## Declaration of interests

DM is a member of COVID-19 Vaccine Strategy Taskforce, NZ Government; COVID-19 Vaccine Strategy Scientific and Technical Advisory Group, NZ Government; Advisory Group, Vaccine Alliance Aotearoa New Zealand (VAANZ); COVID-19 Expert Advisory Network, NZ Ministry of Health; and an independent member of the Clinical Trials Steering Committee, University of Oxford COVID-19 Vaccine trials. CRS received COVID-19 related grant funding from the NZ Health Research Council, NZ Ministry of Business, Innovation and Employment, Chief Scientist Office Scotland, UK National Institute for Health Research and UK Medical Research Council. JdL, NF and TN received COVID-19 related grant funding from the NZ Health Research Council, and NZ Ministry of Business, Innovation and Employment. BN and DW received COVID-19 related grant funding from the NZ Health Research Council. DW received grant funding from the Ministry of Business, Innovation and Employment as part of the Te Pūnaha Matatini COVID-19 Modelling Programme. SJ received COVID-19 related grant funding from the NZ Ministry of Business, Innovation and Employment. SJ and JdL's institute (ESR) received funding from the New Zealand Ministry of Health to undertake national infectious disease surveillance.

## Declaration of Competing Interest

The authors declare no conflict of interest.

## Appendix Supplementary materials

Image, application 1
