# Supplementary material for: COVID-19 vaccine strategies for Aotearoa New Zealand: a mathematical modelling study
Source: Lancet Reg Health West Pac. 2021 Aug 19;15:100256. doi: 10.1016/j.lanwpc.2021.100256 (PMC8375363; doi:10.1016/j.lanwpc.2021.100256)
Supplement: Supplementary file 1 [file mmc1.docx]

## Supplementary Materials

**Table S1: Parameters used in the model.**

| **Parameter** | **Value** | **Source (Reference)** |
| --- | --- | --- |
| Basic reproduction number (R_0_) | 2·5 | Assumed in reported ranges.^1-3^ |
| Presymptomatic infectious period (*t_p_*) | 2 days | ^4,5^ |
| Latent period (not infectious) (1/σ) | 3·8 days | Incubation period (5·8 days^6,7^) – presymptomatic infectious period (2 days). |
| Infectious period for both *Is(s)* and *Id(s)* compartments (*t_d_* and *t_s_*) | 7·2 days | Average from symptom onset to isolation.^8^ |
| New Zealand (NZ)/Māori and Pasifika population counts | 5,000,000/  1,224,140 | ^9^ |
| Age group proportions of NZ (*N_i_*) | 0·125, 0·128, 0·141, 0·139, 0·126, 0·128, 0·106, 0·107 | ^9^ |
| Relative infectiousness of subclinical cases compared with clinical cases (*f*) | 0·5 | For the same period.^10,11^ The infectious durations of subclinical and clinical cases may differ. |
| Age stratified death rates (%) | 0·0016, 0·007, 0·031, 0·084, 0·16, 0·595, 1·93, 5·48 | Estimated infection fatality rate.^12^ |
| Age stratified death rates for Māori and Pasifika populations (%) | 0·01, 0·01, 0·109, 0·11, 1·22, 1·23, 7·20, 8·69 | Extrapolated and combined from the estimated infection fatality rate in.^13^ |
| Average hospitalisation period | 8·9 days | EpiSurv.^14^ |
| Hospitalisation rates (all cases without vaccination) | 0·012, 0·017, 0·016, 0·048, 0·059, 0·091, 0·102, 0·24 | Age-group rates.^14^ Assumed double rates for Māori and Pasifika.^15^ |
| Relative susceptibility (*u_i_*) | 0·4, 0·38, 0·79, 0·86, 0·8, 0·82, 0·88, 0·74 | Age-group details.^10^ |
| Clinical disease rates of infections (ρ_i_) | 0·29, 0·21, 0·27, 0·33, 0·4, 0·49, 0·63, 0·69 | Age-group details.^10^ |
| Age distribution of imported cases | 0·026, 0·029, 0·327, 0·190, 0·108, 0·137, 0·127, 0·056 | EpiSurv.^14^ |

Note: Age-group related parameters are listed in the order of increasing age i.e {0–9,10–19,20–29…,60–69,70+}. Age-stratified death rates of the combined Māori and Pasifika population are combined using their age-stratified population sizes and their corresponding separate rates estimated in.^13^ The death rates for age group 70+ of the whole New Zealand (NZ) or Māori/Pasifika population are combined linearly from the death rates and population sizes (*N_i_*) of groups 70–79 and 80+.

**Table S2: Results of vaccine scenarios from a two-year open border simulation (R_0_=4·5) with ten daily cases introduced to the community and vaccination allowed for 16-plus people**

| **Vaccine scenarios (*e_d_*/*e_i_*, uptake, & R_0_)** | **Vaccine strategies** | **Peak active cases** | **Total community cases** | **Peak hosps.** | **Total hosps.** | **Total deaths** |
| --- | --- | --- | --- | --- | --- | --- |
| 95/90% uniform – 79·8% coverage | n/a | 47,200 | 315,000 | 888 | 4,540 | 209 |
| 95/90% uniform | minimise R_eff_ | 86,400 | 612,000 | 3,760 | 19,800 | 2,840 |
| 70% coverage | high-risk | 166,000 | 1,440,000 | 3,660 | 28,700 | 1,470 |
| 95/90% uniform | minimise R_eff_ | 184,000 | 1,660,000 | 10,000 | 76,200 | 10,000 |
| 60% coverage | high-risk | 313,000 | 2,160,000 | 7,690 | 51,500 | 2,550 |
| 95/90% uniform | minimise R_eff_ | **361,000** | **2,560,000** | 19,500 | 131,000 | 15,300 |
| 50% coverage | high-risk | 508,000 | 2,780,000 | **14,900** | **82,300** | **3,830** |
| 95/80% uniform – 79·8% coverage | n/a | 97,200 | 750,000 | 1,690 | 9,370 | 484 |
| 95/80% uniform | minimise R_eff_ | 150,000 | 1,290,000 | 6,110 | 39,100 | 5,780 |
| 70% coverage | high-risk | 237,000 | 2,060,000 | 4,390 | 34,000 | 1,920 |
| 95/80% uniform | minimise R_eff_ | 282,000 | 2,450,000 | 13,000 | 98,900 | 13,000 |
| 60% coverage | high-risk | 403,000 | 2,760,000 | 8,520 | 55,000 | 2,890 |
| 95/80% uniform | minimise R_eff_ | **467,000** | **3,140,000** | 22,000 | 138,000 | 16,200 |
| 50% coverage | high-risk | 602,000 | 3,290,000 | **15,600** | **83,300** | **3,940** |
| 95/70% uniform – 79·8% coverage | n/a | 178,000 | 1,630,000 | 2,610 | 17,800 | 1,080 |
| 95/70% uniform | minimise R_eff_ | 248,000 | 2,300,000 | 8,350 | 62,700 | 9,380 |
| 70% coverage | high-risk | 334,000 | 2,780,000 | 5,180 | 38,500 | 2,340 |
| 95/70% uniform | minimise R_eff_ | 400,000 | 3,170,000 | 15,600 | 110,000 | 14,500 |
| 60% coverage | high-risk | 504,000 | 3,310,000 | 9,240 | 56,500 | 3,050 |
| 95/90% uniform | minimise R_eff_ | **576,000** | **3,630,000** | 24,100 | 141,000 | 16,700 |
| 70% coverage | high-risk | 694,000 | 3,710,000 | **16,100** | **82,800** | **3,900** |
| 95/60% uniform – 79·8% coverage | n/a | 295,000 | 2,760,000 | 3,530 | 27,000 | 1,850 |
| 95/60% uniform | minimise R_eff_ | 373,000 | 3,220,000 | 10,500 | 78,100 | 11,800 |
| 70% coverage | high-risk | 447,000 | 3,390,000 | 5,910 | 40,500 | 2,550 |
| 95/60% uniform | minimise R_eff_ | 523,000 | 3,700,000 | 17,900 | 114,000 | 15,200 |
| 60% coverage | high-risk | 607,000 | 3,730,000 | 9,810 | 56,400 | 3,050 |
| 90/80% uniform – 79·8% coverage | n/a | 110,000 | 885,000 | 2,960 | 16,300 | 1,190 |
| 90/80% uniform | minimise R_eff_ | 168,000 | 1,470,000 | 8,030 | 53,300 | 7,600 |
| 70% coverage | high-risk | 254,000 | 2,180,000 | 6,840 | 53,700 | 4,130 |
| 90/80% uniform | minimise R_eff_ | 305,000 | 2,590,000 | 15,700 | 119,000 | 15,200 |
| 60% coverage | high-risk | 422,000 | 2,840,000 | 12,000 | 79,200 | 5,760 |
| 90/70% uniform – 79·8% coverage | n/a | 203,000 | 1,880,000 | 4,670 | 32,200 | 2,610 |
| 90/70% uniform | minimise R_eff_ | 275,000 | 2,520,000 | 10,800 | 82,400 | 11,700 |
| 70% coverage | high-risk | 358,000 | 2,920,000 | 8,190 | 61,000 | 4,940 |
| 90/70% uniform | minimise R_eff_ | 427,000 | 3,280,000 | 18,500 | 130,000 | 16,600 |
| 60% coverage | high-risk | 527,000 | 3,390,000 | 12,900 | 80,600 | 6,000 |
| 90/60% uniform – 79·8% coverage | n/a | 329,000 | 2,980,000 | 6,250 | 47,500 | 4,150 |
| 90/60% uniform | minimise R_eff_ | 405,000 | 3,380,000 | 13,500 | 98,700 | 14,000 |
| 70% coverage | high-risk | 476,000 | 3,500,000 | 9,270 | 63,200 | 5,250 |
| 90/60% uniform | minimise R_eff_ | 552,000 | 3,770,000 | 21,000 | 133,000 | 17,200 |
| 60% coverage | high-risk | 631,000 | 3,800,000 | 13,600 | 79,100 | 5,910 |
| 80/70% uniform – 79·8% coverage | n/a | 262,000 | 2,410,000 | 10,700 | 77,400 | 7,570 |
| 80/70% uniform | minimise R_eff_ | 336,000 | 2,940,000 | 17,300 | 132,000 | 17,100 |
| 70% coverage | high-risk | 412,000 | 3,180,000 | 15,900 | 116,000 | 11,400 |
| 80/70% uniform | minimise R_eff_ | 484,000 | 3,490,000 | 25,500 | 174,000 | 21,400 |
| 60% coverage | high-risk | 574,000 | 3,550,000 | 21,800 | 135,000 | 12,800 |
| 80/60% uniform – 79·8% coverage | n/a | 401,000 | 3,350,000 | 13,300 | 98,800 | 10,100 |
| 80/60% uniform | minimise R_eff_ | 475,000 | 3,640,000 | 20,500 | 145,000 | 19,000 |
| 70% coverage | high-risk | 537,000 | 3,700,000 | 17,400 | 115,000 | 11,500 |
| 80/60% uniform | minimise R_eff_ | 610,000 | 3,920,000 | 27,800 | 171,000 | 21,400 |
| 60% coverage | high-risk | 681,000 | 3,930,000 | 22,300 | 129,000 | 12,200 |
| 70/50% uniform – 79·8% coverage | n/a | 614,000 | 4,020,000 | 24,000 | 151,000 | 16,800 |
| 70/50% uniform | minimise R_eff_ | **676,000** | 4,160,000 | 31,000 | 186,000 | 23,800 |
| 60% coverage | high-risk | 718,000 | 4,160,000 | **27,500** | **158,000** | **17,300** |

Note: Maximum vaccination coverage for each age group is now 100% (no limit). The lowest values of five measures for each scenario are in bold.

Note: *e_i_* is the VE of reducing infection. *e_d_* is the VE of preventing disease. The peak and total community cases do not count imported cases (7,300 cases). The peak hospitalisations, total hospitalisations, and total deaths include hospitalised and death cases from the 7,300 imported cases, which are estimated as 444 total hospitalisations and 49·6 deaths when the imported cases are assumed to be not vaccinated. A scenario of “95/70% uniform, 80% coverage” means that the vaccine has uniform effects across age groups with 95% disease prevention reduction and 70% infection reduction, and the uptake is 80% coverage of total population. Maximum vaccination coverage for each age group is now 100% (no limit). The herd immunity threshold is not achievable – no hybrid scenario, where the vaccine has poor infection reduction. Targeted vaccine strategies: (minimise R_eff_) Targeting of younger (socialised) age groups to minimise R_eff_; (high-risk) Groups susceptible to hospitalisation and death; (hybrid) Strategy targeting both younger age groups to achieve the herd immunity threshold and high-risk groups; and (n/a) all strategies are identical as the uptake level is at maximum 100%. Results are rounded to third significant number. The lowest values for each scenario are bold.

**Table S3: Results of various vaccine scenarios from a two-year simulation (R_0_=6) with daily ten cases introduced to the community and vaccination allowed all age groups**

| **Vaccine scenarios (*e_d_*/*e_i_* & uptake)** | **Vaccine strategies** | **Peak active cases** | **Total community cases** | **Peak hosps.** | **Total hosps.** | **Total deaths** |
| --- | --- | --- | --- | --- | --- | --- |
| 95/90% uniform – 100% coverage | n/a | 168 | 8,250 | 9 | 685 | 74 |
| 95/90% uniform | minimise R_eff_ | **424** | **29,200** | **30** | **2,250** | **362** |
| 90% coverage | high-risk | 24,700 | 440,000 | 571 | 9,520 | 705 |
|  | hybrid | 700 | 50,000 | 33 | 2,320 | 296 |
| 95/90% uniform | minimise R_eff_ | **29,200** | **732,000** | **1,940** | 43,500 | 7,190 |
| 80% coverage | high-risk | 147,000 | 1,250,000 | 3,400 | **28,100** | **1,830** |
| 95/90% uniform | minimise R_eff_ | **166,000** | **1,700,000** | 9,700 | 91,000 | 13,200 |
| 70% coverage | high-risk | 296,000 | 1,960,000 | **6,640** | **44,400** | **2,830** |
| 95/80% uniform – 100% coverage | n/a | 771 | 60,100 | 18 | 1,340 | 139 |
| 95/80% uniform | minimise R_eff_ | **15,300** | **704,000** | **654** | 25,800 | 4,260 |
| 90% coverage | high-risk | 69,500 | 1,210,000 | 1,240 | **19,000** | **1,600** |
| 95/80% uniform | minimise R_eff_ | **128,000** | **1,890,000** | 5,930 | 75,000 | 12,200 |
| 80% coverage | high-risk | 243,000 | 2,200,000 | **4,420** | **37,000** | **2,710** |
| 95/80% uniform | minimise R_eff_ | **313,000** | **2,710,000** | 13,400 | 103,000 | 14,900 |
| 70% coverage | high-risk | 426,000 | 2,840,000 | **7,750** | **50,100** | **3,460** |
| 95/70% uniform – 100% coverage | n/a | 70,300 | 1,710,000 | 877 | 18,100 | 1,910 |
| 95/70% uniform | minimise R_eff_ | **158,000** | **2,420,000** | 4,290 | 56,100 | 9,010 |
| 90% coverage | high-risk | 205,000 | 2,580,000 | **2,670** | **29,500** | **2,710** |
| 95/70% uniform | minimise R_eff_ | **301,000** | **3,060,000** | 9,980 | 86,900 | 14,000 |
| 80% coverage | high-risk | 396,000 | 3,200,000 | **5,600** | **41,600** | **3,240** |
| 95/70% uniform | minimise R_eff_ | **490,000** | **3,540,000** | 16,800 | 108,000 | 15,800 |
| 70% coverage | high-risk | 579,000 | 3,570,000 | **8,630** | **51,100** | **3,650** |
| 95/60% uniform – 100% coverage | n/a | 262,000 | 3,000,000 | 2,410 | 24,000 | 2,660 |
| 95/60% uniform | minimise R_eff_ | **364,000** | **3,430,000** | 7,010 | 58,000 | 9,220 |
| 90% coverage | high-risk | 397,000 | 3,500,000 | **4,000** | **31,600** | **3,010** |
| 95/60% uniform | minimise R_eff_ | **500,000** | **3,780,000** | 13,000 | 87,500 | 14,100 |
| 80% coverage | high-risk | 572,000 | 3,850,000 | **6,540** | **41,200** | **3,270** |
| 95/60% uniform | minimise R_eff_ | **667,000** | 4,070,000 | 19,300 | 108,000 | 15,900 |
| 70% coverage | high-risk | 731,000 | **4,050,000** | **9,190** | **49,400** | **3,530** |
| 90/80% uniform – 100% coverage | n/a | 2,380 | 164,000 | 89 | 5,400 | 551 |
| 90/80% uniform | minimise R_eff_ | **39,700** | **1,210,000** | **2,070** | 54,900 | 8,020 |
| 90% coverage | high-risk | 91,400 | 1,530,000 | 2,900 | **43,000** | **4,190** |
| 90/80% uniform | minimise R_eff_ | **164,000** | **2,180,000** | 8,780 | 103,000 | 15,200 |
| 80% coverage | high-risk | 269,000 | 2,430,000 | **7,930** | **67,800** | **6,200** |
| 90/80% uniform | minimise R_eff_ | **349,000** | **2,880,000** | 17,100 | 130,000 | 17,800 |
| 70% coverage | high-risk | 453,000 | 2,980,000 | **12,700** | **83,900** | **7,390** |
| 90/70% uniform – 100% coverage | n/a | 119,000 | 2,150,000 | 2,920 | 45,100 | 4,850 |
| 90/70% uniform | minimise R_eff_ | **213,000** | **2,750,000** | 7,320 | 82,600 | 11,700 |
| 90% coverage | high-risk | 253,000 | 2,860,000 | **5,960** | **59,600** | **6,100** |
| 90/70% uniform | minimise R_eff_ | **351,000** | **3,260,000** | 13,800 | 114,000 | 16,800 |
| 80% coverage | high-risk | 436,000 | 3,380,000 | **10,100** | **73,200** | **6,950** |
| 90/70% uniform | minimise R_eff_ | **531,000** | **3,670,000** | 21,000 | 134,000 | 18,400 |
| 70% coverage | high-risk | 613,000 | 3,690,000 | **14,000** | **83,100** | **7,510** |
| 90/60% uniform – 100% coverage | n/a | 321,000 | 3,230,000 | 5,800 | 51,400 | 5,780 |
| 90/60% uniform | minimise R_eff_ | **420,000** | **3,600,000** | 11,000 | 84,900 | 12,200 |
| 90% coverage | high-risk | 451,000 | 3,660,000 | **8,150** | **60,200** | **6,350** |
| 90/60% uniform | minimise R_eff_ | **548,000** | **3,900,000** | 17,200 | 113,000 | 16,800 |
| 80% coverage | high-risk | 616,000 | 3,960,000 | **11,500** | **70,100** | **6,760** |
| 90/60% uniform | minimise R_eff_ | **708,000** | 4,140,000 | 23,500 | 131,000 | 18,400 |
| 70% coverage | high-risk | 766,000 | **4,130,000** | **14,500** | **78,000** | **7,090** |
| 80/70% uniform – 100% coverage | n/a | 235,000 | 2,810,000 | 11,100 | 119,000 | 13,200 |
| 80/70% uniform | minimise R_eff_ | **333,000** | **3,250,000** | 16,900 | 152,000 | 19,100 |
| 90% coverage | high-risk | 361,000 | 3,310,000 | **15,900** | **135,000** | **14,800** |
| 80/70% uniform | minimise R_eff_ | **450,000** | **3,580,000** | 23,800 | 178,000 | 23,400 |
| 80% coverage | high-risk | 526,000 | 3,690,000 | **22,000** | **148,000** | **15,900** |
| 80/70% uniform | minimise R_eff_ | **617,000** | 3,890,000 | 31,400 | 193,000 | 24,600 |
| 70% coverage | high-risk | 684,000 | 3,890,000 | **26,900** | **156,000** | **16,500** |
| 80/60% uniform – 100% coverage | n/a | 441,000 | 3,590,000 | 15,400 | 115,000 | 13,300 |
| 80/60% uniform | minimise R_eff_ | **537,000** | **3,880,000** | 21,000 | 142,000 | 18,400 |
| 90% coverage | high-risk | 561,000 | 3,920,000 | **18,800** | **124,000** | **14,000** |
| 80/60% uniform | minimise R_eff_ | **648,000** | **4,100,000** | 27,200 | 166,000 | 22,300 |
| 80% coverage | high-risk | 707,000 | 4,140,000 | **23,200** | **133,000** | **14,600** |
| 80/60% uniform | minimise R_eff_ | **790,000** | 4,280,000 | 33,700 | 181,000 | 23,800 |
| 70% coverage | high-risk | 838,000 | **4,260,000** | **26,800** | **140,000** | **14,900** |
| 70/60% uniform – 100% coverage | n/a | 560,000 | 3,860,000 | 28,300 | 187,000 | 22,000 |
| 70/60% uniform | minimise R_eff_ | **651,000** | **4,090,000** | 33,800 | 208,000 | 25,900 |
| 90% coverage | high-risk | 670,000 | 4,120,000 | **32,200** | **195,000** | **22,800** |
| 70/60% uniform | minimise R_eff_ | **746,000** | **4,250,000** | 39,200 | 223,000 | 28,400 |
| 80% coverage | high-risk | 800,000 | 4,290,000 | **37,200** | **202,000** | **23,300** |
| 70/50% uniform – 100% coverage | n/a | 734,000 | 4,210,000 | 29,300 | 165,000 | 19,800 |
| 70/50% uniform | minimise R_eff_ | **819,000** | 4,390,000 | 34,300 | 183,000 | 23,300 |
| 90% coverage | high-risk | 831,000 | 4,390,000 | **32,100** | **171,000** | **20,200** |

Note: Maximum vaccination coverage for each age group is now 100% (no limit). The lowest values of five measures for each scenario are in bold.

**Table S4: Results of various vaccine scenarios from a two-year simulation (R_0_=6) with daily ten cases introduced to the community and vaccination allowed for 12-plus people**

| **Vaccine scenarios (*e_d_*/*e_i_*, uptake, & R_0_)** | **Vaccine strategies** | **Peak active cases** | **Total community cases** | **Peak hosps.** | **Total hosps.** | **Total deaths** |
| --- | --- | --- | --- | --- | --- | --- |
| 95/90% uniform – 84·9% coverage | n/a | 73,100 | 830,000 | 1,660 | 17,800 | 1,260 |
| 95/90% uniform | minimise R_eff_ | 91,700 | 1,020,000 | 3,690 | 38,100 | 5,230 |
| 80% coverage | high-risk | 138,000 | 1,240,000 | 3,160 | 27,400 | 1,820 |
| 95/90% uniform | minimise R_eff_ | 186,000 | 1,700,000 | 9,700 | 82,400 | 12,500 |
| 70% coverage | high-risk | 292,000 | 1,960,000 | 6,490 | 44,000 | 2,810 |
| 95/90% uniform | minimise R_eff_ | 360,000 | 2,430,000 | 18,800 | 122,000 | 15,900 |
| 60% coverage | high-risk | 487,000 | 2,590,000 | 12,100 | 67,200 | 3,940 |
| 95/90% uniform | minimise R_eff_ | 579,000 | 3,020,000 | 29,300 | 153,000 | 18,100 |
| 50% coverage | high-risk | 713,000 | 3,130,000 | 20,600 | 97,300 | 5,300 |
| 95/80% uniform – 84·9% coverage | n/a | 145,000 | 1,760,000 | 2,580 | 28,100 | 2,240 |
| 95/80% uniform | minimise R_eff_ | 177,000 | 2,010,000 | 5,670 | 56,100 | 7,920 |
| 80% coverage | high-risk | 236,000 | 2,200,000 | 4,240 | 36,400 | 2,720 |
| 95/80% uniform | minimise R_eff_ | 315,000 | 2,690,000 | 12,900 | 97,300 | 14,500 |
| 70% coverage | high-risk | 420,000 | 2,850,000 | 7,580 | 49,800 | 3,450 |
| 95/80% uniform | minimise R_eff_ | 514,000 | 3,270,000 | 21,800 | 128,000 | 16,800 |
| 60% coverage | high-risk | 624,000 | 3,340,000 | 12,900 | 69,200 | 4,240 |
| 95/80% uniform | minimise R_eff_ | 723,000 | 3,690,000 | 31,400 | 155,000 | 18,500 |
| 50% coverage | high-risk | 842,000 | 3,730,000 | 21,000 | 96,000 | 5,250 |
| 95/70% uniform – 84·9% coverage | n/a | 295,000 | 2,940,000 | 3,980 | 35,400 | 3,020 |
| 95/70% uniform | minimise R_eff_ | 336,000 | 3,130,000 | 8,150 | 66,200 | 9,460 |
| 80% coverage | high-risk | 392,000 | 3,210,000 | 5,480 | 41,100 | 3,250 |
| 95/70% uniform | minimise R_eff_ | 486,000 | 3,550,000 | 15,800 | 103,000 | 15,400 |
| 70% coverage | high-risk | 574,000 | 3,600,000 | 8,480 | 50,900 | 3,640 |
| 95/70% uniform | minimise R_eff_ | 677,000 | 3,900,000 | 24,200 | 129,000 | 17,100 |
| 60% coverage | high-risk | 764,000 | 3,890,000 | 13,500 | 67,900 | 4,170 |
| 95/70% uniform | minimise R_eff_ | 862,000 | 4,150,000 | 33,000 | 154,000 | 18,600 |
| 50% coverage | high-risk | 962,000 | 4,150,000 | 21,300 | 93,200 | 4,960 |
| 90/80% uniform – 84·9% coverage | n/a | 170,000 | 2,030,000 | 5,190 | 56,400 | 5,360 |
| 90/80% uniform | minimise R_eff_ | 205,000 | 2,270,000 | 8,660 | 85,900 | 11,300 |
| 80% coverage | high-risk | 263,000 | 2,430,000 | 7,730 | 67,300 | 6,210 |
| 90/80% uniform | minimise R_eff_ | 348,000 | 2,870,000 | 16,600 | 126,000 | 17,500 |
| 70% coverage | high-risk | 447,000 | 3,000,000 | 12,500 | 83,900 | 7,420 |
| 90/80% uniform | minimise R_eff_ | 544,000 | 3,380,000 | 26,200 | 156,000 | 19,800 |
| 60% coverage | high-risk | 648,000 | 3,430,000 | 18,700 | 104,000 | 8,540 |
| 90/70% uniform – 84·9% coverage | n/a | 340,000 | 3,160,000 | 7,910 | 66,600 | 6,600 |
| 90/70% uniform | minimise R_eff_ | 381,000 | 3,320,000 | 12,200 | 96,300 | 12,800 |
| 80% coverage | high-risk | 433,000 | 3,390,000 | 9,970 | 72,900 | 6,970 |
| 90/70% uniform | minimise R_eff_ | 527,000 | 3,680,000 | 20,100 | 130,000 | 18,100 |
| 70% coverage | high-risk | 609,000 | 3,720,000 | 13,800 | 83,200 | 7,530 |
| 90/70% uniform | minimise R_eff_ | 710,000 | 3,980,000 | 28,600 | 154,000 | 19,800 |
| 60% coverage | high-risk | 792,000 | 3,970,000 | 19,200 | 99,200 | 8,190 |
| 80/70% uniform – 84·9% coverage | n/a | 441,000 | 3,530,000 | 18,800 | 142,000 | 15,400 |
| 80/70% uniform | minimise R_eff_ | 480,000 | 3,660,000 | 23,100 | 166,000 | 20,600 |
| 80% coverage | high-risk | 524,000 | 3,700,000 | 21,700 | 148,000 | 15,900 |
| 80/70% uniform | minimise R_eff_ | 612,000 | 3,910,000 | 30,600 | 191,000 | 24,400 |
| 70% coverage | high-risk | 683,000 | 3,930,000 | 26,800 | 156,000 | 16,600 |
| 80/70% uniform | minimise R_eff_ | 778,000 | 4,120,000 | 38,700 | 208,000 | 25,700 |
| 60% coverage | high-risk | 850,000 | 4,100,000 | 32,600 | 168,000 | 17,200 |
| 80/60% uniform – 84·9% coverage | n/a | 633,000 | 4,050,000 | 20,900 | 129,000 | 14,400 |
| 80/60% uniform | minimise R_eff_ | 670,000 | 4,140,000 | 25,700 | 154,000 | 19,700 |
| 80% coverage | high-risk | 706,000 | 4,160,000 | 23,000 | 133,000 | 14,600 |
| 80/60% uniform | minimise R_eff_ | 785,000 | 4,300,000 | 32,700 | 177,000 | 23,500 |
| 70% coverage | high-risk | 839,000 | 4,290,000 | 26,700 | 140,000 | 15,000 |
| 80/60% uniform | minimise R_eff_ | 926,000 | 4,430,000 | 39,800 | 194,000 | 24,600 |
| 60% coverage | high-risk | 978,000 | 4,390,000 | 31,600 | 151,000 | 15,400 |
| 70/50% uniform – 84·9% coverage | n/a | 888,000 | 4,460,000 | 33,800 | 174,000 | 20,400 |
| 70/50% uniform | minimise R_eff_ | 1,010,000 | 4,600,000 | 44,200 | 211,000 | 27,500 |
| 70% coverage | high-risk | 1,040,000 | 4,580,000 | 38,700 | 181,000 | 20,800 |

Note: Maximum vaccination coverage for each age group is now 100% (no limit). The lowest values of five measures for each scenario are in bold.

**Table S5: Results of various vaccine scenarios from a two-year simulation (R_0_=6) with daily ten cases introduced to the community and vaccination allowed for 16-plus people**

| **Vaccine scenarios (*e_d_*/*e_i_*, uptake, & R_0_)** | **Vaccine strategies** | **Peak active cases** | **Total community cases** | **Peak hosps.** | **Total hosps.** | **Total deaths** |
| --- | --- | --- | --- | --- | --- | --- |
| 95/90% uniform – 79·8% coverage | n/a | 143,000 | 1,220,000 | 3,250 | 25,200 | 1,610 |
| 95/90% uniform | minimise R_eff_ | 215,000 | 1,710,000 | 10,300 | 75,700 | 11,300 |
| 70% coverage | high-risk | 296,000 | 1,940,000 | 6,570 | 43,300 | 2,730 |
| 95/90% uniform | minimise R_eff_ | 365,000 | 2,420,000 | 18,700 | 119,000 | 15,600 |
| 60% coverage | high-risk | 487,000 | 2,570,000 | 11,800 | 65,000 | 3,820 |
| 95/90% uniform | minimise R_eff_ | 579,000 | 3,020,000 | 29,300 | 152,000 | 18,000 |
| 50% coverage | high-risk | 712,000 | 3,120,000 | 20,100 | 94,800 | 5,150 |
| 95/80% uniform – 79·8% coverage | n/a | 241,000 | 2,140,000 | 4,330 | 34,000 | 2,450 |
| 95/80% uniform | minimise R_eff_ | 336,000 | 2,660,000 | 12,800 | 89,600 | 13,300 |
| 70% coverage | high-risk | 420,000 | 2,830,000 | 7,600 | 49,200 | 3,370 |
| 95/80% uniform | minimise R_eff_ | 516,000 | 3,270,000 | 21,500 | 125,000 | 16,600 |
| 60% coverage | high-risk | 622,000 | 3,320,000 | 12,600 | 67,300 | 4,140 |
| 95/80% uniform | minimise R_eff_ | 723,000 | 3,690,000 | 31,400 | 155,000 | 18,500 |
| 50% coverage | high-risk | 840,000 | 3,720,000 | 20,500 | 93,700 | 5,110 |
| 95/70% uniform – 79·8% coverage | n/a | 396,000 | 3,190,000 | 5,550 | 40,100 | 3,120 |
| 95/70% uniform | minimise R_eff_ | 499,000 | 3,550,000 | 15,300 | 96,100 | 14,300 |
| 70% coverage | high-risk | 573,000 | 3,590,000 | 8,480 | 50,600 | 3,580 |
| 95/70% uniform | minimise R_eff_ | 678,000 | 3,900,000 | 23,900 | 128,000 | 17,000 |
| 60% coverage | high-risk | 761,000 | 3,890,000 | 13,100 | 66,100 | 4,090 |
| 95/90% uniform | minimise R_eff_ | 862,000 | 4,150,000 | 33,000 | 154,000 | 18,600 |
| 70% coverage | high-risk | 959,000 | 4,140,000 | 20,700 | 90,900 | 4,840 |
| 95/60% uniform – 79·8% coverage | n/a | 572,000 | 3,860,000 | 6,480 | 40,400 | 3,220 |
| 95/60% uniform | minimise R_eff_ | 670,000 | 4,080,000 | 17,300 | 96,500 | 14,500 |
| 70% coverage | high-risk | 728,000 | 4,080,000 | 9,070 | 48,900 | 3,470 |
| 95/60% uniform | minimise R_eff_ | 829,000 | 4,280,000 | 25,900 | 127,000 | 17,000 |
| 60% coverage | high-risk | 890,000 | 4,250,000 | 13,400 | 63,500 | 3,840 |
| 90/80% uniform – 79·8% coverage | n/a | 268,000 | 2,360,000 | 7,850 | 62,300 | 5,650 |
| 90/80% uniform | minimise R_eff_ | 365,000 | 2,850,000 | 16,700 | 118,000 | 16,300 |
| 70% coverage | high-risk | 447,000 | 2,990,000 | 12,500 | 82,900 | 7,300 |
| 90/80% uniform | minimise R_eff_ | 546,000 | 3,380,000 | 26,000 | 154,000 | 19,500 |
| 60% coverage | high-risk | 646,000 | 3,420,000 | 18,400 | 102,000 | 8,430 |
| 90/70% uniform – 79·8% coverage | n/a | 437,000 | 3,370,000 | 10,100 | 71,500 | 6,790 |
| 90/70% uniform | minimise R_eff_ | 538,000 | 3,680,000 | 19,700 | 124,000 | 17,200 |
| 70% coverage | high-risk | 607,000 | 3,710,000 | 13,800 | 82,700 | 7,470 |
| 90/70% uniform | minimise R_eff_ | 711,000 | 3,970,000 | 28,500 | 153,000 | 19,700 |
| 60% coverage | high-risk | 789,000 | 3,960,000 | 18,900 | 97,600 | 8,100 |
| 90/60% uniform – 79·8% coverage | n/a | 617,000 | 3,970,000 | 11,400 | 69,300 | 6,720 |
| 90/60% uniform | minimise R_eff_ | 710,000 | 4,170,000 | 21,800 | 121,000 | 17,200 |
| 70% coverage | high-risk | 764,000 | 4,160,000 | 14,400 | 77,600 | 7,040 |
| 90/60% uniform | minimise R_eff_ | 861,000 | 4,330,000 | 30,200 | 149,000 | 19,500 |
| 60% coverage | high-risk | 918,000 | 4,300,000 | 19,000 | 91,200 | 7,480 |
| 80/70% uniform – 79·8% coverage | n/a | 528,000 | 3,690,000 | 21,900 | 146,000 | 15,700 |
| 80/70% uniform | minimise R_eff_ | 620,000 | 3,920,000 | 30,400 | 187,000 | 23,800 |
| 70% coverage | high-risk | 681,000 | 3,930,000 | 26,800 | 156,000 | 16,500 |
| 80/70% uniform | minimise R_eff_ | 778,000 | 4,120,000 | 38,700 | 207,000 | 25,600 |
| 60% coverage | high-risk | 846,000 | 4,100,000 | 32,300 | 167,000 | 17,200 |
| 80/60% uniform – 79·8% coverage | n/a | 709,000 | 4,160,000 | 23,100 | 133,000 | 14,500 |
| 80/60% uniform | minimise R_eff_ | 793,000 | 4,310,000 | 32,300 | 174,000 | 22,900 |
| 70% coverage | high-risk | 838,000 | 4,290,000 | 26,800 | 140,000 | 14,900 |
| 80/60% uniform | minimise R_eff_ | 926,000 | 4,420,000 | 39,800 | 194,000 | 24,600 |
| 60% coverage | high-risk | 976,000 | 4,390,000 | 31,300 | 150,000 | 15,300 |
| 70/50% uniform – 79·8% coverage | n/a | 946,000 | 4,520,000 | 35,600 | 176,000 | 20,500 |
| 70/50% uniform | minimise R_eff_ | 1,010,000 | 4,600,000 | 43,900 | 209,000 | 27,100 |
| 60% coverage | high-risk | 1,040,000 | 4,580,000 | 38,700 | 181,000 | 20,700 |

Note: Maximum vaccination coverage for each age group is now 100% (no limit). The lowest values of five measures for each scenario are in bold.

**Table S6: Results of various vaccine scenarios from a two-year simulation (R_0_=2·5) with daily ten cases introduced to the community and vaccination allowed for all age groups**

| **Vaccine scenarios (*e_d_*/*e_i_* & uptake)** | **Vaccine strategies** | **Peak active cases** | **Total community cases** | **Peak hosps.** | **Total hosps.** | **Total deaths** |
| --- | --- | --- | --- | --- | --- | --- |
| 95/90% uniform – 90% coverage | n/a | 144 | 5,860 | 9 | 707 | 76 |
| 95/90% uniform | minimise R_eff_ | 190 | 9,720 | 13 | 1,000 | 127 |
| 80% coverage | high-risk | 188 | 9,660 | 10 | 800 | 83 |
|  | hybrid | 188 | 9,660 | 10 | 800 | 83 |
| 95/90% uniform | minimise R_eff_ | 310 | 19,800 | 21 | 1,590 | 219 |
| 70% coverage | high-risk | 1,120 | 72,000 | 39 | 2,450 | 192 |
|  | hybrid | 458 | 31,700 | 20 | 1,530 | 133 |
| 95/90% uniform | minimise R_eff_ | 1,310 | 90,700 | 86 | 5,630 | 707 |
| 60% coverage | high-risk | 26,200 | 734,000 | 852 | 21,300 | 1,380 |
|  | hybrid | 2,020 | 133,000 | 123 | 7,550 | 867 |
| 95/80% uniform – 90% coverage | n/a | 205 | 11,500 | 10 | 795 | 85 |
| 95/80% uniform | minimise R_eff_ | 279 | 17,800 | 16 | 1,210 | 156 |
| 80% coverage | high-risk | 281 | 18,100 | 12 | 946 | 96 |
|  | hybrid | 281 | 18,100 | 12 | 946 | 96 |
| 95/80% uniform | minimise R_eff_ | 506 | 35,900 | 29 | 2,180 | 305 |
| 70% coverage | high-risk | 3,450 | 201,000 | 103 | 5,330 | 406 |
|  | hybrid | 928 | 66,900 | 38 | 2,630 | 234 |
| 95/80% uniform | minimise R_eff_ | 5,950 | 340,000 | 333 | 17,000 | 2,140 |
| 60% coverage | high-risk | 46,900 | 1,070,000 | 1,380 | 27,800 | 1,860 |
| 95/70% uniform – 90% coverage | n/a | 327 | 22,600 | 12 | 969 | 102 |
| 95/70% uniform | minimise R_eff_ | 483 | 35,300 | 21 | 1,610 | 205 |
| 80% coverage | high-risk | 509 | 37,500 | 17 | 1,280 | 128 |
|  | hybrid | 509 | 37,500 | 17 | 1,280 | 128 |
| 95/70% uniform | minimise R_eff_ | 1,270 | 92,100 | 62 | 4,190 | 599 |
| 70% coverage | high-risk | 11,600 | 531,000 | 300 | 11,900 | 930 |
|  | hybrid | 1,880 | 131,000 | 77 | 4,840 | 601 |
| 95/70% uniform | minimise R_eff_ | 24,300 | 911,000 | 1,200 | 39,400 | 4,940 |
| 60% coverage | high-risk | 76,600 | 1,460,000 | 2,040 | 34,000 | 2,360 |
| 95/60% uniform – 90% coverage | n/a | 697 | 53,600 | 20 | 1,450 | 151 |
| 95/60% uniform | minimise R_eff_ | 1,370 | 102,000 | 47 | 3,160 | 405 |
| 80% coverage | high-risk | 1,600 | 117,000 | 40 | 2,630 | 253 |
|  | hybrid | 1,570 | 115,000 | 43 | 2,830 | 296 |
| 95/60% uniform | minimise R_eff_ | 7,940 | 456,000 | 325 | 16,200 | 2,330 |
| 70% coverage | high-risk | 32,100 | 1,040,000 | 727 | 20,200 | 1,640 |
| 95/60% uniform | minimise R_eff_ | 59,800 | 1,490,000 | 2,530 | 55,200 | 6,950 |
| 60% coverage | high-risk | 115,000 | 1,870,000 | 2,770 | 39,400 | 2,820 |
| 95/50% uniform – 90% coverage | n/a | 3,830 | 258,000 | 78 | 4,480 | 458 |
| 95/50% uniform | minimise R_eff_ | 11,300 | 612,000 | 290 | 13,400 | 1,690 |
| 80% coverage | high-risk | 13,400 | 693,000 | 261 | 11,500 | 1,100 |
| 95/50% uniform | minimise R_eff_ | 38,100 | 1,270,000 | 1,290 | 37,000 | 5,250 |
| 70% coverage | high-risk | 68,800 | 1,600,000 | 1,370 | 27,400 | 2,300 |
| 95/40% uniform – 90% coverage | n/a | 29,700 | 1,100,000 | 485 | 15,200 | 1,580 |
| 95/40% uniform | minimise R_eff_ | 49,800 | 1,460,000 | 975 | 24,300 | 2,870 |
| 80% coverage | high-risk | 53,400 | 1,510,000 | 892 | 21,500 | 2,090 |
| 90/80% uniform – 90% coverage | n/a | 220 | 12,600 | 12 | 956 | 101 |
| 90/80% uniform | minimise R_eff_ | 304 | 19,600 | 18 | 1,410 | 175 |
| 80% coverage | high-risk | 311 | 20,300 | 15 | 1,170 | 119 |
|  | hybrid | 311 | 20,300 | 15 | 1,170 | 119 |
| 90/80% uniform | minimise R_eff_ | 599 | 42,600 | 38 | 2,730 | 374 |
| 70% coverage | high-risk | 4,680 | 257,000 | 166 | 8,080 | 662 |
|  | hybrid | 1,030 | 73,100 | 51 | 3,470 | 343 |
| 90/70% uniform – 90% coverage | n/a | 371 | 26,100 | 16 | 1,250 | 130 |
| 90/70% uniform | minimise R_eff_ | 580 | 42,600 | 29 | 2,110 | 264 |
| 80% coverage | high-risk | 619 | 45,700 | 24 | 1,760 | 175 |
|  | hybrid | 618 | 45,700 | 24 | 1,760 | 175 |
| 90/70% uniform | minimise R_eff_ | 1,840 | 128,000 | 98 | 6,210 | 864 |
| 70% coverage | high-risk | 15,400 | 640,000 | 483 | 17,500 | 1,480 |
|  | hybrid | 2,440 | 163,000 | 120 | 7,200 | 934 |
| 90/60% uniform – 90% coverage | n/a | 930 | 70,600 | 31 | 2,190 | 226 |
| 90/60% uniform | minimise R_eff_ | 2,160 | 152,000 | 81 | 5,050 | 614 |
| 80% coverage | high-risk | 2,650 | 181,000 | 80 | 4,770 | 466 |
|  | hybrid | 2,480 | 171,000 | 89 | 5,450 | 636 |
| 90/60% uniform | minimise R_eff_ | 13,100 | 663,000 | 590 | 25,900 | 3,600 |
| 70% coverage | high-risk | 40,200 | 1,170,000 | 1,120 | 28,300 | 2,470 |
| 90/50% uniform – 90% coverage | n/a | 7,390 | 438,000 | 188 | 9,490 | 972 |
| 90/50% uniform | minimise R_eff_ | 19,000 | 862,000 | 553 | 21,500 | 2,530 |
| 80% coverage | high-risk | 21,500 | 932,000 | 531 | 19,600 | 1,940 |
| 90/50% uniform | minimise R_eff_ | 50,000 | 1,450,000 | 1,900 | 47,700 | 6,550 |
| 70% coverage | high-risk | 81,200 | 1,740,000 | 2,000 | 37,100 | 3,330 |
| 90/40% uniform – 90% coverage | n/a | 42,300 | 1,310,000 | 902 | 23,700 | 2,490 |
| 90/40% uniform | minimise R_eff_ | 64,700 | 1,650,000 | 1,520 | 33,100 | 3,750 |
| 80% coverage | high-risk | 68,400 | 1,690,000 | 1,470 | 31,000 | 3,110 |
| 80/70% uniform – 90% coverage | n/a | 525 | 37,500 | 29 | 2,160 | 224 |
| 8070% uniform | minimise R_eff_ | 968 | 69,700 | 57 | 3,950 | 470 |
| 80% coverage | high-risk | 1,100 | 78,400 | 55 | 3,710 | 373 |
|  | hybrid | 1,100 | 78,300 | 55 | 3,710 | 373 |
| 80/70% uniform | minimise R_eff_ | 5,130 | 303,000 | 319 | 16,900 | 2,230 |
| 70% coverage | high-risk | 26,100 | 883,000 | 1,130 | 33,800 | 3,150 |
| 80/60% uniform – 90% coverage | n/a | 2,340 | 159,000 | 104 | 6,300 | 644 |
| 80/60% uniform | minimise R_eff_ | 7,510 | 428,000 | 365 | 18,100 | 2,140 |
| 80% coverage | high-risk | 9,030 | 497,000 | 373 | 17,800 | 1,780 |
| 80/60% uniform | minimise R_eff_ | 29,600 | 1,090,000 | 1,570 | 50,800 | 6,610 |
| 70% coverage | high-risk | 60,100 | 1,450,000 | 2,310 | 49,100 | 4,690 |
| 70/50% uniform – 90% coverage | n/a | 47,100 | 1,340,000 | 2,250 | 56,200 | 5,940 |
| 70/50% uniform | minimise R_eff_ | 70,800 | 1,680,000 | 3,380 | 70,700 | 7,800 |
| 80% coverage | high-risk | 74,600 | 1,720,000 | 3,420 | 69,500 | 7,290 |
| 70/30% uniform ­– 90% coverage | n/a | 184,000 | 2,540,000 | 6,610 | 80,300 | 8,820 |

Note: *e_i_* is the VE of reducing infection. *e_d_* is the VE of preventing disease. The peak and total community cases do not count imported cases (7,300 cases). The peak hospitalisations, total hospitalisations, and total deaths include hospitalised and death cases from the 7,300 imported cases, which are estimated as 444 total hospitalisations and 84 deaths when the imported cases are assumed to be not vaccinated. A scenario of “95/70% uniform, 80% coverage” means that the vaccine has uniform effects across age groups with 95% disease prevention reduction and 70% infection reduction, and the uptake is 80% coverage of total population. The herd immunity threshold is not achievable – no hybrid scenario, where the vaccine has poor infection reduction. Targeted vaccine strategies: (minimise R_eff_) Targeting of younger (socialised) age groups to minimise R_eff_; (high-risk) Groups susceptible to hospitalisation and death; (hybrid) Strategy targeting both younger age groups to achieve the herd immunity threshold and high-risk groups; and (n/a) all strategies are identical as the uptake level is at maximum 90%. Results are rounded to third significant number. The lowest values for each scenario are in bold.

**Table S7: Results of various vaccine scenarios from a two-year simulation (R_0_=2·5) with daily ten cases introduced to the community and vaccination allowed only for people aged at least 12**

| **Vaccine scenarios (*e_d_*/*e_i_* & uptake)** | **Vaccine strategies** | **Peak active cases** | **Total community cases** | **Peak hosps.** | **Total hosps.** | **Total deaths** |
| --- | --- | --- | --- | --- | --- | --- |
| 95/90% uniform – 76·4% coverage | n/a | 233 | 13,500 | 11 | 893 | 90 |
| 95/90% uniform | minimise R_eff_ | 316 | 20,100 | 19 | 1,450 | 188 |
| 70% coverage | high-risk | 991 | 63,700 | 35 | 2,210 | 177 |
|  | hybrid | 429 | 29,300 | 18 | 1,330 | 120 |
| 95/90% uniform | minimise R_eff_ | 1,120 | 77,500 | 69 | 4,600 | 593 |
| 60% coverage | high-risk | 21,000 | 661,000 | 636 | 17,900 | 1,210 |
|  | hybrid | 1,700 | 114,000 | 98 | 6,100 | 688 |
| 95/80% uniform – 76·4% coverage | n/a | 368 | 25,200 | 14 | 1,110 | 109 |
| 95/80% uniform | minimise R_eff_ | 540 | 38,400 | 28 | 2,030 | 267 |
| 70% coverage | high-risk | 2,840 | 169,000 | 85 | 4,530 | 351 |
|  | hybrid | 865 | 62,200 | 33 | 2,270 | 196 |
| 95/80% uniform | minimise R_eff_ | 4,340 | 262,000 | 234 | 12,600 | 1,630 |
| 60% coverage | high-risk | 38,300 | 976,000 | 1,050 | 23,700 | 1,660 |
| 95/70% uniform – 76·4% coverage | n/a | 766 | 56,800 | 24 | 1,680 | 160 |
| 95/70% uniform | minimise R_eff_ | 1,400 | 100,000 | 60 | 3,940 | 526 |
| 70% coverage | high-risk | 9,400 | 462,000 | 243 | 10,400 | 817 |
|  | hybrid | 1,710 | 120,000 | 68 | 4,310 | 535 |
| 95/70% uniform | minimise R_eff_ | 19,200 | 811,000 | 899 | 33,300 | 4,300 |
| 60% coverage | high-risk | 64,500 | 1,360,000 | 1,600 | 29,700 | 2,160 |
| 95/60% uniform – 76·4% coverage | n/a | 3,480 | 228,000 | 82 | 4,670 | 429 |
| 95/60% uniform | minimise R_eff_ | 8,520 | 481,000 | 305 | 14,900 | 2,010 |
| 70% coverage | high-risk | 27,000 | 958,000 | 610 | 18,600 | 1,520 |
| 95/60% uniform | minimise R_eff_ | 51,600 | 1,420,000 | 2,100 | 50,200 | 6,480 |
| 60% coverage | high-risk | 101,000 | 1,790,000 | 2,260 | 35,100 | 2,630 |
| 90/80% uniform – 76·4% coverage | n/a | 419 | 28,900 | 19 | 1,410 | 140 |
| 90/80% uniform | minimise R_eff_ | 641 | 45,600 | 36 | 2,590 | 335 |
| 70% coverage | high-risk | 3,810 | 216,000 | 135 | 6,820 | 568 |
|  | hybrid | 974 | 69,000 | 44 | 2,970 | 291 |
| 90/80% uniform | minimise R_eff_ | 6,620 | 368,000 | 384 | 19,100 | 2,430 |
| 60% coverage | high-risk | 43,900 | 1,050,000 | 1,450 | 30,800 | 2,400 |
| 90/70% uniform – 76·4% coverage | n/a | 1,010 | 73,200 | 36 | 2,470 | 238 |
| 90/70% uniform | minimise R_eff_ | 2,070 | 140,000 | 98 | 6,050 | 792 |
| 70% coverage | high-risk | 12,500 | 566,000 | 393 | 15,500 | 1,320 |
|  | hybrid | 2,480 | 164,000 | 115 | 6,890 | 881 |
| 90/70% uniform | minimise R_eff_ | 25,900 | 960,000 | 1,310 | 42,900 | 5,420 |
| 60% coverage | high-risk | 72,800 | 1,450,000 | 2,180 | 38,400 | 3,080 |
| 90/60% uniform – 76·4% coverage | n/a | 6,090 | 364,000 | 175 | 9,030 | 855 |
| 90/60% uniform | minimise R_eff_ | 13,700 | 685,000 | 554 | 23,900 | 3,130 |
| 70% coverage | high-risk | 34,300 | 1,100,000 | 950 | 26,300 | 2,330 |
| 90/60% uniform | minimise R_eff_ | 62,700 | 1,550,000 | 2,800 | 60,800 | 7,660 |
| 60% coverage | high-risk | 112,000 | 1,880,000 | 3,030 | 45,000 | 3,720 |
| 80/70% uniform – 76·4% coverage | n/a | 2,270 | 149,000 | 107 | 6,340 | 625 |
| 80/70% uniform | minimise R_eff_ | 5,690 | 329,000 | 326 | 16,800 | 2,100 |
| 70% coverage | high-risk | 21,700 | 805,000 | 937 | 30,800 | 2,890 |
| 80/70% uniform | minimise R_eff_ | 42,900 | 1,250,000 | 2,510 | 65,300 | 7,960 |
| 60% coverage | high-risk | 91,700 | 1,640,000 | 3,720 | 59,800 | 5,400 |
| 80/60% uniform – 76·4% coverage | n/a | 17,000 | 784,000 | 682 | 27,400 | 2,720 |
| 80/60% uniform | minimise R_eff_ | 30,100 | 1,100,000 | 1,480 | 47,700 | 5,930 |
| 70% coverage | high-risk | 52,700 | 1,380,000 | 2,030 | 46,800 | 4,500 |
| 80/60% uniform | minimise R_eff_ | 87,800 | 1,820,000 | 4,540 | 83,600 | 10,200 |
| 60% coverage | high-risk | 136,000 | 2,080,000 | 5,010 | 68,500 | 6,320 |

Note: The smallest values in each scenario are bold. Age group coverage is limited to be not greater than 90%. Total attainable vaccine coverage is, therefore, 76·4%.

**Table S8: Results of various vaccine scenarios from a two-year simulation (R_0_=2·5) where vaccine is only allowed for people aged at least 16 – daily ten cases introduced to the community**

| **Vaccine scenarios (*e_d_*/*e_i_*, uptake, & R_0_)** | **Vaccine strategies** | **Peak active cases** | **Total community cases** | **Peak hosps.** | **Total hosps.** | **Total deaths** |
| --- | --- | --- | --- | --- | --- | --- |
| 95/90% uniform – 71·8% coverage | n/a | 392 | 25,900 | 16 | 1,220 | 111 |
| 95/90% uniform | minimise R_eff_ | **434** | **29,100** | **19** | **1,390** | **138** |
| 70% coverage | high-risk | 991 | 63,700 | 35 | 2,210 | 177 |
|  | hybrid | 490 | 33,400 | 20 | 1,420 | 125 |
| 95/90% uniform | minimise R_eff_ | **1,310** | **88,200** | **76** | **4,860** | **627** |
| 60% coverage | high-risk | 24,200 | 695,000 | 731 | 18,800 | 1,260 |
|  | hybrid | 1,790 | 117,000 | 99 | 6,080 | 709 |
| 95/90% uniform | minimise R_eff_ | **28,500** | **881,000** | **1,750** | 48,700 | 5,550 |
| 50% coverage | high-risk | 102,000 | 1,510,000 | 3,570 | **47,500** | **2,770** |
| 95/80% uniform – 71·8% coverage | n/a | 833 | 58,300 | 28 | 1,900 | 165 |
| 95/80% uniform | minimise R_eff_ | **910** | **63,500** | **34** | **2,260** | 232 |
| 70% coverage | high-risk | 2,840 | 169,000 | 85 | 4,530 | 351 |
|  | hybrid | 1,010 | 70,300 | 36 | 2,370 | **227** |
| 95/80% uniform | minimise R_eff_ | **5,150** | **298,000** | **261** | **13,500** | 1,750 |
| 60% coverage | high-risk | 40,800 | 993,000 | 1,120 | 24,100 | **1,680** |
| 95/80% uniform | minimise R_eff_ | **55,900** | **1,310,000** | **3,070** | 63,900 | 7,290 |
| 50% coverage | high-risk | 134,000 | 1,830,000 | 4,300 | **52,400** | **3,140** |
| 95/70% uniform – 71·8% coverage | n/a | 3,180 | 199,000 | 85 | 4,700 | 392 |
| 95/70% uniform | minimise R_eff_ | **3,760** | **231,000** | **113** | **6,100** | **609** |
| 70% coverage | high-risk | 9,400 | 462,000 | 243 | 10,400 | 817 |
| 95/70% uniform | minimise R_eff_ | **20,100** | **827,000** | **907** | 32,700 | 4,240 |
| 60% coverage | high-risk | 66,000 | 1,370,000 | 1,640 | **29,800** | **2,160** |
| 95/70% uniform | minimise R_eff_ | **93,600** | **1,750,000** | **4,580** | 75,900 | 8,700 |
| 50% coverage | high-risk | 172,000 | 2,160,000 | 5,060 | **56,600** | **3,470** |
| 95/60% uniform – 71·8% coverage | n/a | 15,400 | 718,000 | 349 | 13,900 | 1,180 |
| 95/60% uniform | minimise R_eff_ | **51,900** | **1,420,000** | **2,090** | 49,700 | 6,430 |
| 60% coverage | high-risk | 101,000 | 1,790,000 | 2,280 | **35,100** | **2,630** |
| 90/80% uniform – 71·8% coverage | n/a | 1,080 | 73,600 | 41 | 2,700 | 243 |
| 90/80% uniform | minimise R_eff_ | **7,560** | **404,000** | **421** | **20,100** | 2,550 |
| 60% coverage | high-risk | 46,100 | 1,060,000 | 1,520 | 31,200 | **2,420** |
| 90/70% uniform – 71·8% coverage | n/a | 4,870 | 286,000 | 156 | 8,020 | 706 |
| 90/70% uniform | minimise R_eff_ | **26,700** | **970,000** | **1,320** | 42,300 | 5,360 |
| 60% coverage | high-risk | 74,000 | 1,460,000 | 2,220 | **38,500** | **3,090** |
| 90/60% uniform – 71·8% coverage | n/a | 21,700 | 886,000 | 606 | 21,300 | 1,920 |
| 90/60% uniform | minimise R_eff_ | **62,800** | **1,550,000** | **2,750** | 59,600 | 7,550 |
| 60% coverage | high-risk | 112,000 | 1,880,000 | 3,040 | **45,100** | **3,720** |
| 80/70% uniform – 71·8% coverage | n/a | 11,300 | 553,000 | 498 | 21,400 | 2,030 |
| 80/70% uniform | minimise R_eff_ | **43,300** | **1,250,000** | **2,520** | 65,200 | 7,940 |
| 60% coverage | high-risk | 92,300 | 1,640,000 | 3,740 | **59,800** | **5,400** |
| 80/60% uniform – 71·8% coverage | n/a | 38,900 | 1,220,000 | 1,510 | 41,500 | 4,040 |
| 80/60% uniform | minimise R_eff_ | **87,800** | **1,820,000** | **4,500** | 83,000 | 10,100 |
| 60% coverage | high-risk | 136,000 | 2,080,000 | 5,000 | **68,500** | **6,320** |
| 70/50% uniform | minimise R_eff_ | **123,000** | **2,160,000** | **5,680** | 89,200 | 9,760 |
| 70% coverage | high-risk | 133,000 | 2,220,000 | 5,830 | **86,900** | **8,910** |
| 70/50% uniform | minimise R_eff_ | **264,000** | **2,930,000** | 14,300 | 145,000 | 16,600 |
| 50% coverage | high-risk | 322,000 | 3,050,000 | **14,100** | **124,000** | **11,700** |

Note: The smallest values in each scenario are bold. Maximum vaccination coverage for each age group is 90%.

**Table S9: Results of vaccine scenarios with reduced effectiveness (50%) in older age groups (60+) from a two-year simulation (R_0_=2·5) – daily 10 cases introduced to the community**

| **Vaccine scenarios (*e_d_*/*e_i_* & uptake)** | **Vaccine strategies** | **Peak active cases** | **Total community cases** | **Peak hosps.** | **Total hosps.** | **Total deaths** |
| --- | --- | --- | --- | --- | --- | --- |
| 95/90% varied | minimise R_eff_ | **238** | **13,600** | 20 | 1,520 | 234 |
| 80% coverage | high-risk | 254 | 15,100 | **18** | **1,430** | **208** |
|  | hybrid | 254 | 15,100 | **18** | **1,430** | **208** |
| 95/90% varied | minimise R_eff_ | **368** | **24,300** | **29** | **2,210** | **348** |
| 70% coverage | high-risk | 1,930 | 119,000 | 107 | 6,130 | 857 |
|  | hybrid | 728 | 51,500 | 47 | 3,350 | 462 |
| 95/80% varied | minimise R_eff_ | **360** | **24,200** | 26 | 2,020 | 318 |
| 80% coverage | high-risk | 397 | 27,400 | **25** | **1,890** | **282** |
|  | hybrid | 397 | 27,400 | **25** | **1,890** | **282** |
| 95/80% varied | minimise R_eff_ | **627** | **45,000** | **44** | **3,230** | **520** |
| 70% coverage | high-risk | 6,330 | 335,000 | 320 | 15,000 | 2,150 |
|  | hybrid | 1,530 | 107,000 | 93 | 6,010 | 866 |
| 90/80% varied | minimise R_eff_ | **403** | **27,400** | 32 | 2,380 | 367 |
| 80% coverage | high-risk | 452 | 31,500 | **31** | **2,290** | **332** |
|  | hybrid | 452 | 31,500 | **31** | **2,290** | **332** |
| 90/80% varied | minimise R_eff_ | **752** | **53,700** | **56** | **4,000** | **628** |
| 70% coverage | high-risk | 8,560 | 422,000 | 473 | 20,700 | 2,890 |
|  | hybrid | 1,700 | 116,000 | 111 | 7,060 | 1,020 |
| 90/70% varied | minimise R_eff_ | **849** | **62,200** | **58** | **4,120** | 651 |
| 80% coverage | high-risk | 1,080 | 78,700 | 63 | 4,300 | **638** |
|  | hybrid | 1,080 | 78,700 | 63 | 4,300 | **638** |
| 90/70% varied | minimise R_eff_ | **2,700** | **178,000** | **177** | **10,600** | **1,690** |
| 70% coverage | high-risk | 25,300 | 901,000 | 1,270 | 40,000 | 5,680 |
|  | hybrid | 2,910 | 190,000 | 189 | 11,100 | 1,770 |

Note: The model assumption is that there are daily ten cases introduced to the community. The smallest values in each scenario are bold. In all scenarios, vaccines are assumed to have half effectiveness on people aged 60 and over. The reduction of effectiveness is assumed to the same for both vaccine effectiveness on infection reduction and disease prevention.

**Table S10: Two-year open border modelling results (10 imported cases/day) with means and standard deviations when the contact matrix is added with a uniform distribution (R_0_=4·5)**

| **Vaccine scenarios (*e_d_*/*e_i_* & uptake)** | **Vaccine strategies** | **Peak active cases** | **Total community cases** | **Peak hosps.** | **Total hosps.** | **Total deaths** |
| --- | --- | --- | --- | --- | --- | --- |
| 90/80% uniform | minimise R_eff_ | **910±55** | **66,920±3,860** | **52.8±3.9** | **3,700 ±240** | **550±42** |
| 90% coverage | high-risk | 12,070±4,260 | 443,070±82,106 | 350±120 | 11,500±2,040 | 1,030±181 |
|  | hybrid | 1,440±155 | 102,500±9,860 | 69±9 | 4,560 ±529 | 600±84 |

Note: *e_i_* is the vaccine effectiveness on infection reduction and *e_d_* is the vaccine effectiveness on disease prevention. The lowest values for each scenario are in bold.

**Table S11:** **Comparison of cases, hospitalisations and deaths in Māori and Pasifika populations (R_0_=4·5) – 10 external cases introduced to the community per day**

| **Vaccine scenarios**  **(*e_d/_e_i_* & uptake)** | **Vaccine strategies** | **Peak active cases** | **Total community cases** | **Peak hosps.** | **Total hosps.** | **Total deaths** |
| --- | --- | --- | --- | --- | --- | --- |
| 95/90% uniform | minimise R_eff_ | 199 | 2,720 | 19 | 374 | 53 |
| 90% coverage | high-risk | 205 | 2,770 | 16 | 326 | 41 |
|  | hybrid | 205 | 2,770 | 16 | 326 | 41 |
| 95/90% uniform | minimise R_eff_ | 534 | 8,420 | 40 | 661 | 102 |
| 80% coverage | high-risk | 1,060 | 13,000 | 44 | 585 | 49 |
|  | hybrid | 560 | 8,380 | 30 | 491 | 47 |
| 95/90% uniform | minimise R_eff_ | 4,610 | 49,700 | 265 | 2,490 | 385 |
| 70% coverage | high-risk | 14,200 | 150,000 | 556 | 4,570 | 150 |
| 95/80% uniform | minimise R_eff_ | 459 | 7,690 | 25 | 445 | 55 |
| 90% coverage | high-risk | 498 | 8,210 | 23 | 411 | 47 |
|  | hybrid | 498 | 8,210 | 23 | 411 | 47 |
| 95/80% uniform | minimise R_eff_ | 2,210 | 28,100 | 97 | 1,140 | 151 |
| 80% coverage | high-risk | 3,340 | 36,700 | 100 | 1,020 | 70 |
| 95/80% uniform | minimise R_eff_ | 17,700 | 175,000 | 800 | 6,190 | 881 |
| 70% coverage | high-risk | 25,200 | 284,000 | 830 | 7,460 | 278 |
| 90/80% uniform | minimise R_eff_ | 620 | 9,910 | 41 | 662 | 78 |
| 90% coverage | high-risk | 675 | 10,600 | 39 | 625 | 69 |
|  | hybrid | 675 | 10,600 | 39 | 625 | 69 |
| 90/80% uniform | minimise R_eff_ | 3,450 | 39,200 | 181 | 1,830 | 222 |
| 80% coverage | high-risk | 4,730 | 48,900 | 186 | 1,700 | 130 |
| 90/80% uniform | minimise R_eff_ | 22,300 | 226,000 | 1,270 | 9,700 | 1,300 |
| 70% coverage | high-risk | 28,500 | 320,000 | 1,230 | 11,000 | 617 |
| 90/70% uniform | minimise R_eff_ | 5,090 | 55,900 | 192 | 1,810 | 191 |
| 90% coverage | high-risk | 5,280 | 57,600 | 181 | 1,700 | 165 |
| 90/70% uniform | minimise R_eff_ | 21,000 | 195,000 | 792 | 5,710 | 533 |
| 80% coverage | high-risk | 21,900 | 208,000 | 740 | 5,380 | 405 |
| 90/70% uniform | minimise R_eff_ | 49,100 | 543,000 | 2,350 | 21,100 | 2,820 |
| 70% coverage | high-risk | 51,000 | 562,000 | 1,880 | 17,300 | 1,130 |
| 80/70% uniform | minimise R_eff_ | 17,400 | 154,000 | 1,100 | 7,780 | 826 |
| 90% coverage | high-risk | 17,800 | 158,000 | 1,050 | 7,440 | 746 |
| 80/70% uniform | minimise R_eff_ | 37,700 | 381,000 | 2,460 | 18,100 | 1,790 |
| 80% coverage | high-risk | 37,800 | 388,000 | 2,330 | 17,400 | 1,600 |
| 80/70% uniform | minimise R_eff_ | 65,400 | 674,000 | 4,400 | 38,900 | 4,620 |
| 70% coverage | high-risk | 65,400 | 676,000 | 3,850 | 34,300 | 3,000 |
| 80/60% uniform | minimise R_eff_ | 54,900 | 598,000 | 2,960 | 25,900 | 2,770 |
| 90% coverage | high-risk | 55,100 | 600,000 | 2,810 | 24,600 | 2,500 |
| 80/60% uniform | minimise R_eff_ | 73,100 | 744,000 | 3,830 | 33,200 | 3,360 |
| 80% coverage | high-risk | 73,700 | 747,000 | 3,660 | 31,700 | 3,030 |
| 80/60% uniform | minimise R_eff_ | 100,000 | 864,000 | 5,650 | 44,200 | 5,300 |
| 70% coverage | high-risk | 99,500 | 862,000 | 4,900 | 38,600 | 3,460 |

Note: Vaccine coverage for each age group has no limit (100% maximum). When the herd immunity threshold is achieved, the outcomes are close to the numbers for the whole New Zealand because their magnitudes are mainly dependent on the number of daily imported cases and the R_eff_ of the simulated population. Both simulations have the same daily imported cases. The R_eff_ of these populations is slightly different from the R_eff_ of the whole NZ in the same scenario due to different age group distribution. The high-risk and hybrid strategies may have the same allocations, e.g. the first vaccine scenario. The lowest values for each scenario are in bold.

**Table S12:** **Comparison of cases, hospitalisations and deaths in Māori and Pasifika populations (R_0_=4·5) – 10 external cases introduced to the community per day. Vaccine is not allowed for children aged under 12.**

| **Vaccine scenarios**  **(*e_d/_e_i_* & uptake)** | **Vaccine strategies** | **Peak active cases** | **Total community cases** | **Peak hosps.** | **Total hosps.** | **Total deaths** |
| --- | --- | --- | --- | --- | --- | --- |
| 95/90% uniform – 74% coverage | n/a | 6,760 | 62,200 | 221 | 1,750 | 82 |
| 95/90% uniform | minimise R_eff_ | 9,260 | 88,000 | 346 | 2,690 | 159 |
| 70% coverage | high-risk | 11,200 | 103,000 | 384 | 2,850 | 110 |
| 95/90% uniform | minimise R_eff_ | 25,500 | 282,000 | 1,420 | 12,500 | 1,700 |
| 60% coverage | high-risk | 39,100 | 384,000 | 1,480 | 12,900 | 420 |
| 95/80% uniform – 74% coverage | n/a | 13,600 | 132,000 | 402 | 3,090 | 139 |
| 95/80% uniform | minimise R_eff_ | 18,900 | 195,000 | 661 | 5,120 | 305 |
| 70% coverage | high-risk | 21,100 | 218,000 | 670 | 5,250 | 207 |
| 95/80% uniform | minimise R_eff_ | 44,600 | 485,000 | 2,170 | 19,900 | 2,940 |
| 60% coverage | high-risk | 53,100 | 519,000 | 1,770 | 15,300 | 578 |
| 95/80% uniform – 74% coverage | n/a | 16,200 | 160,000 | 649 | 4,840 | 299 |
| 95/80% uniform | minimise R_eff_ | 22,400 | 235,000 | 1,050 | 8,050 | 620 |
| 70% coverage | high-risk | 24,400 | 257,000 | 1,030 | 8,040 | 463 |
| 95/80% uniform | minimise R_eff_ | 49,900 | 530,000 | 2,890 | 26,200 | 3,640 |
| 60% coverage | high-risk | 56,700 | 550,000 | 2,390 | 21,000 | 1,210 |
| 95/80% uniform – 74% coverage | n/a | 35,600 | 393,000 | 1,280 | 10,700 | 737 |
| 95/80% uniform | minimise R_eff_ | 45,700 | 508,000 | 1,910 | 16,800 | 1,600 |
| 70% coverage | high-risk | 46,700 | 518,000 | 1,700 | 15,000 | 993 |
| 95/80% uniform | minimise R_eff_ | 78,400 | 740,000 | 3,910 | 32,800 | 4,530 |
| 60% coverage | high-risk | 79,800 | 734,000 | 2,960 | 24,700 | 1,560 |
| 95/80% uniform – 74% coverage | n/a | 50,600 | 552,000 | 3,020 | 25,900 | 2,330 |
| 95/80% uniform | minimise R_eff_ | 61,000 | 646,000 | 3,810 | 33,500 | 3,280 |
| 70% coverage | high-risk | 61,800 | 651,000 | 3,620 | 31,800 | 2,800 |
| 95/80% uniform | minimise R_eff_ | 93,500 | 813,000 | 6,220 | 49,800 | 6,150 |
| 60% coverage | high-risk | 92,900 | 808,000 | 5,220 | 42,500 | 3,630 |
| 95/80% uniform – 74% coverage | n/a | 86,000 | 815,000 | 4,220 | 35,300 | 3,280 |
| 95/80% uniform | minimise R_eff_ | 96,000 | 857,000 | 5,020 | 40,300 | 4,070 |
| 70% coverage | high-risk | 96,600 | 858,000 | 4,710 | 37,800 | 3,420 |
| 95/80% uniform | minimise R_eff_ | 126,000 | 938,000 | 7,210 | 50,300 | 6,260 |
| 60% coverage | high-risk | 124,000 | 935,000 | 6,020 | 42,800 | 3,680 |

Note: Vaccine coverage for each age group has no limit (100% maximum). Maximum attainable vaccine coverage is 74%.


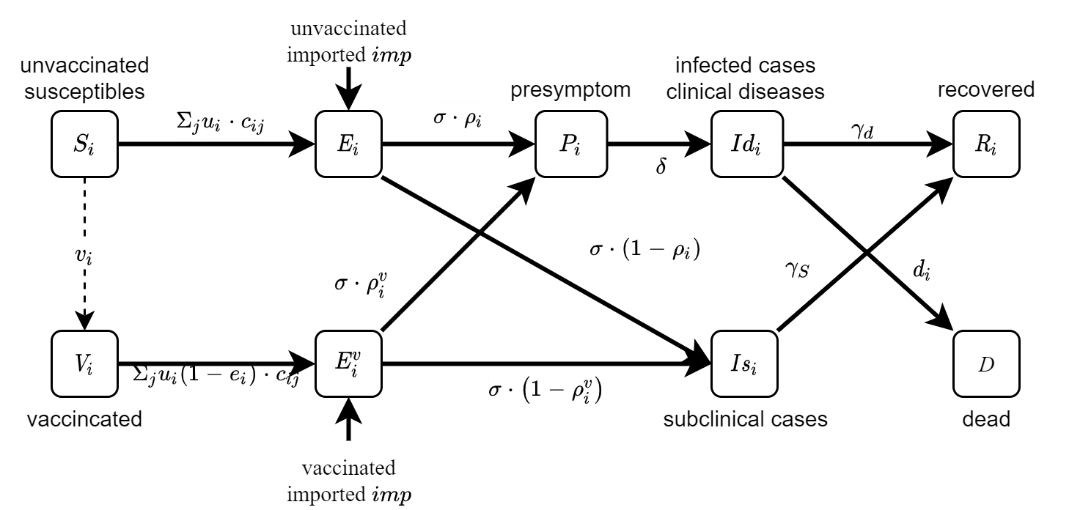


**Figure S1: The age-stratified SEIR model for COVID-19.**

Note: The transition parameters *δ, γ_d_, γ_S_, σ* are the inverse of the average periods in the corresponding former compartments. Specifically, σ=1/3·8 with 3·8 days as the latent period,*δ=1/t_p_* with *t_p_* as the average presymtomatic infectious period, $\gamma_{d}=(1-d_{i})/t_{d}$ and *γ_S_=1/t_S_* with *t_d_* and *t_s_* as the average infectious periods of clinical and subclinical cases respectively (Supplemental Table S1), *d_i_* is the death rate of age group *i*. Refer to Method section for the description of compartments and other parameters.


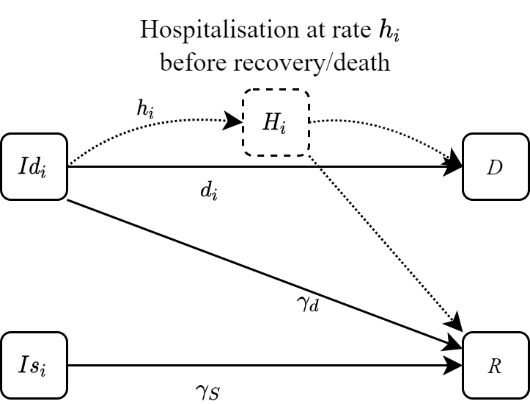


**Figure S2: Modelling hospitalisations.**

Note: *d_i_* and *h_i_* is the death rate and hospitalisation rate of age group *i*.


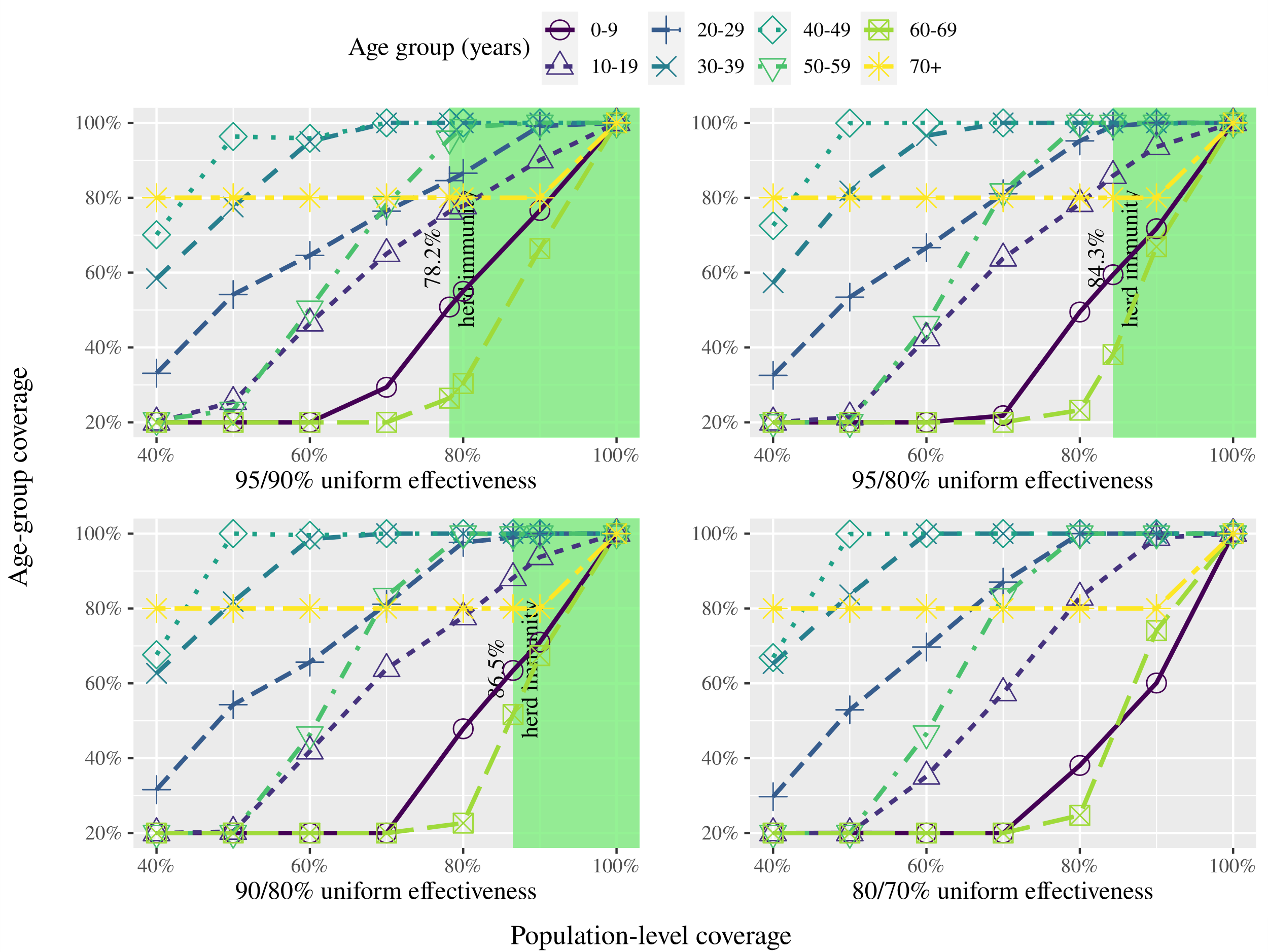


**Figure S3: Age-group allocations of vaccine strategy 1 at various VE scenarios (R_0_=4·5)**

Note: Vaccine allocations of the spread-minimising strategy (strategy 1) at fixed uptake levels and minimal uptake level required for the herd immunity threshold (border lines of the green areas). A vaccine has two values of effectiveness: disease prevention and infection reduction. The effectiveness of a vaccine is called “uniform” if their effectiveness is equal across age groups.


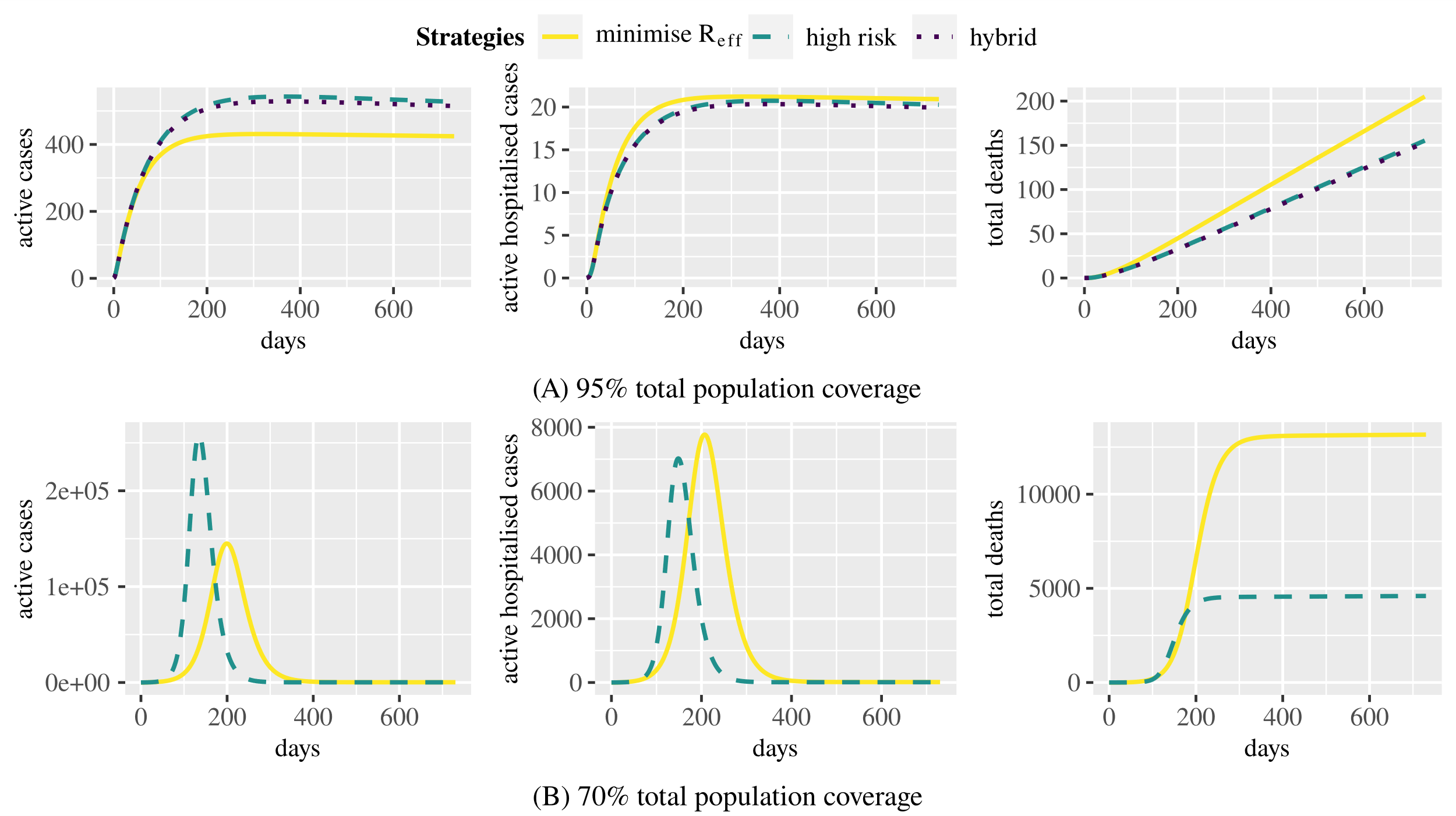


**Figure S4: The changes of active cases and hospitalised cases over the two-year period of simulations (R_0_=4·5) – 90/80% uniform effectiveness with (A) 95% coverage – and (B) 70% coverage**


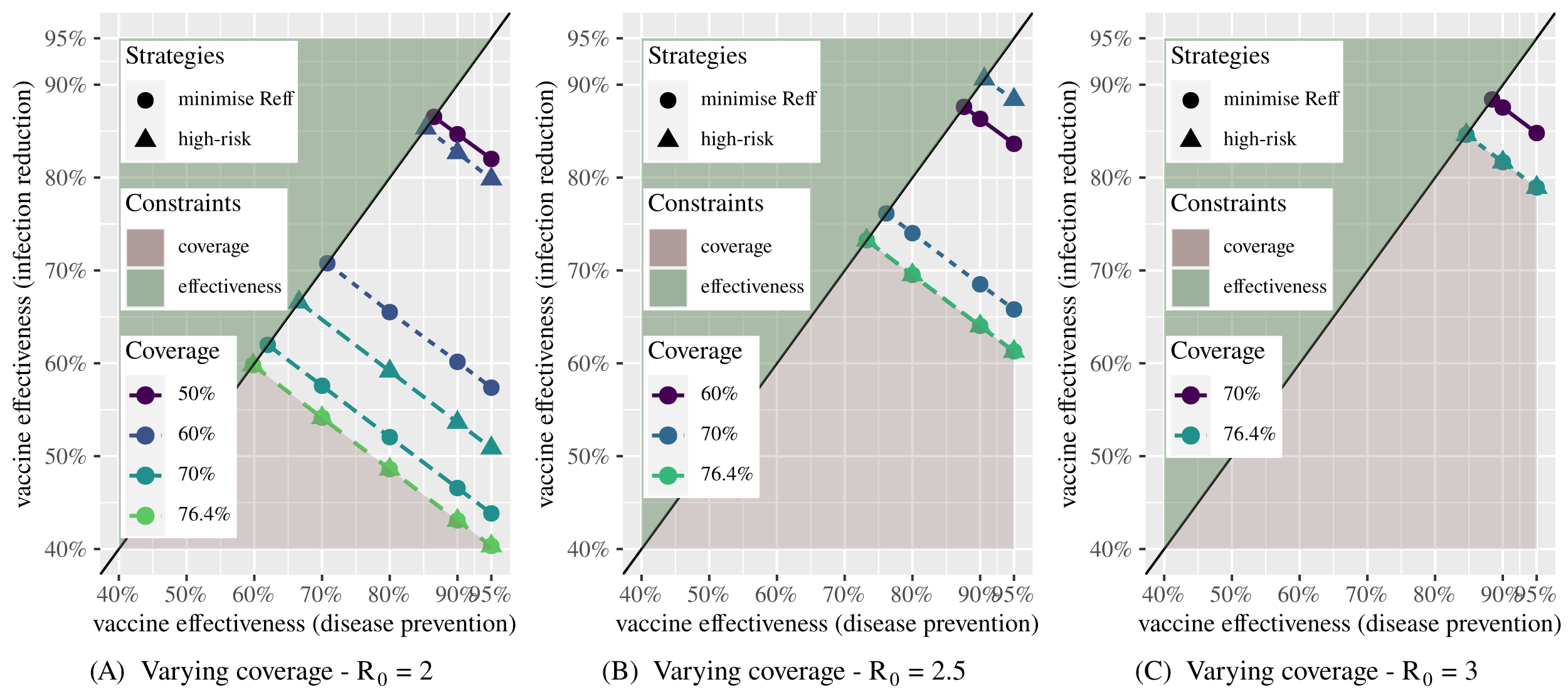


**Figure S5: Vaccine effectiveness and New Zealand population vaccine uptake requirements for the herd immunity threshold (R_0_=2–3·5) with vaccination allowed for individuals aged at least 12**

Note: The minimal VE of infection reduction and disease prevention for the herd immunity threshold at multiple vaccine uptake levels given a fixed R_0_=2 (A), 2·5 (B), and 3 (C). Vaccine coverage for each age group is limited to 90% maximum. HIT is not achievable for R_0_=3·5 in this scenario.

**
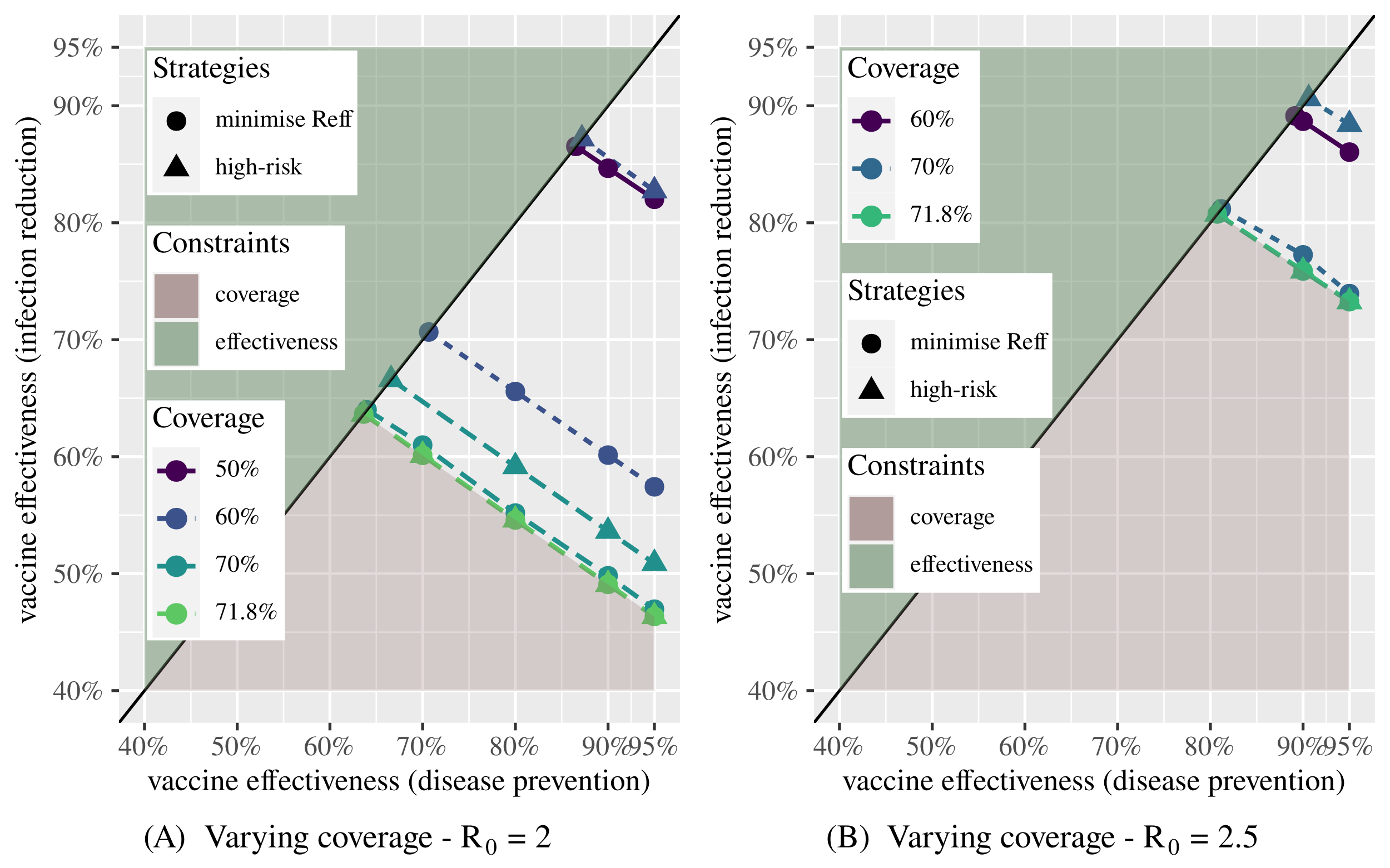
**

**Figure S6: Vaccine effectiveness and New Zealand population vaccine uptake requirements for the herd immunity threshold (R_0_=2–3·5) with vaccination allowed for individuals aged at least 16**

Note: The minimal VE of infection reduction and disease prevention for the herd immunity threshold at multiple vaccine uptake levels given a fixed R_0_=2 (A) and 2·5 (B). Vaccine coverage for each age group is limited to 90% maximum. HIT is not achievable for R_0_=3 and 3·5 in this scenario.


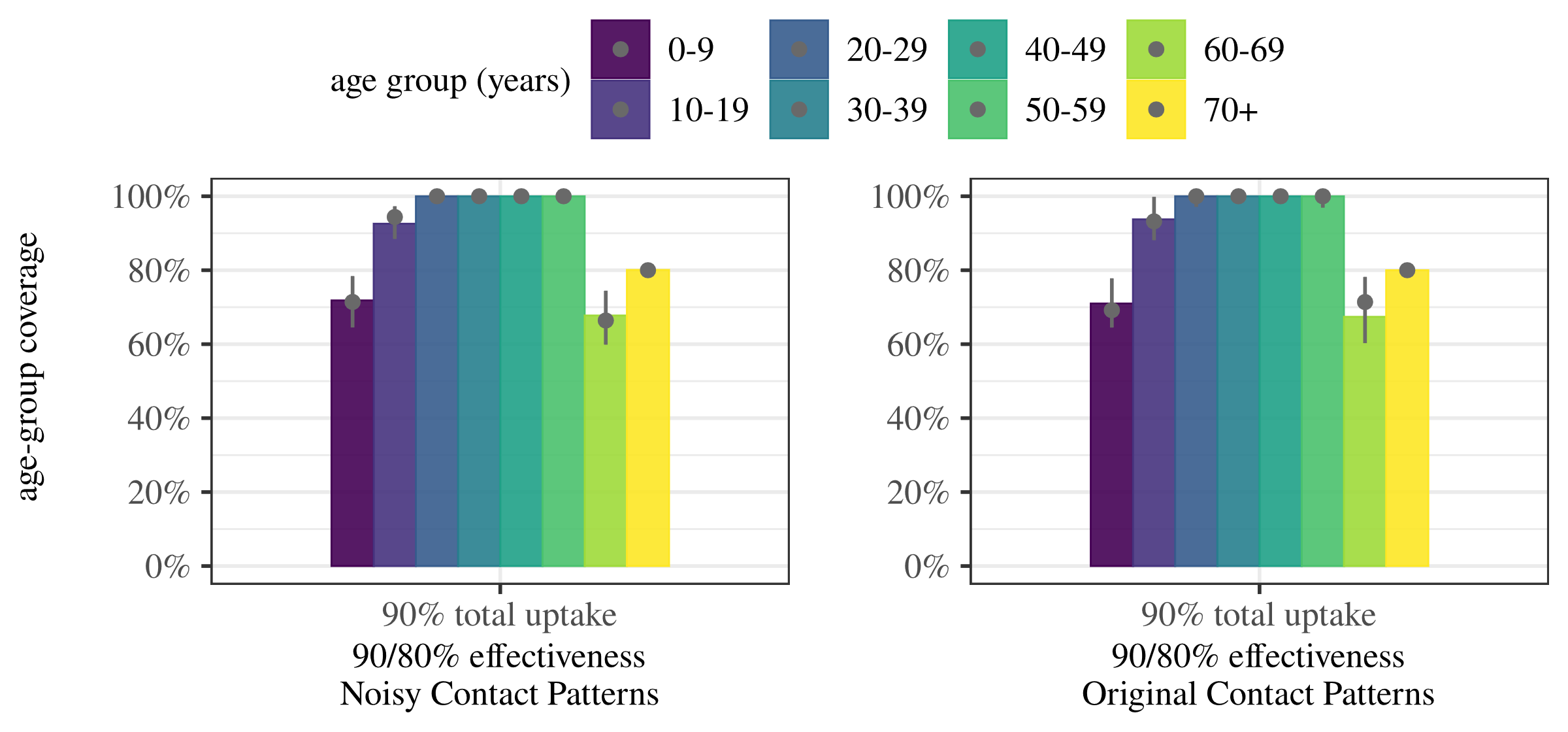


**Figure S7: Comparison of the changes of vaccine allocations (using the spread-minimising strategy) caused by slightly changing the contact matrix and by 50 times of optimising R_eff_ with the original contact matrix (and different random seeds).**

Note: The variations of vaccine allocations caused by adding noise to the contact matrix are generally smaller than the deviations caused by different random seeds. 90/80% effectiveness means the vaccine has 90% effectiveness on preventing disease and 80% effectiveness on reducing infection. Vaccine effectiveness here is considered equal across age groups.


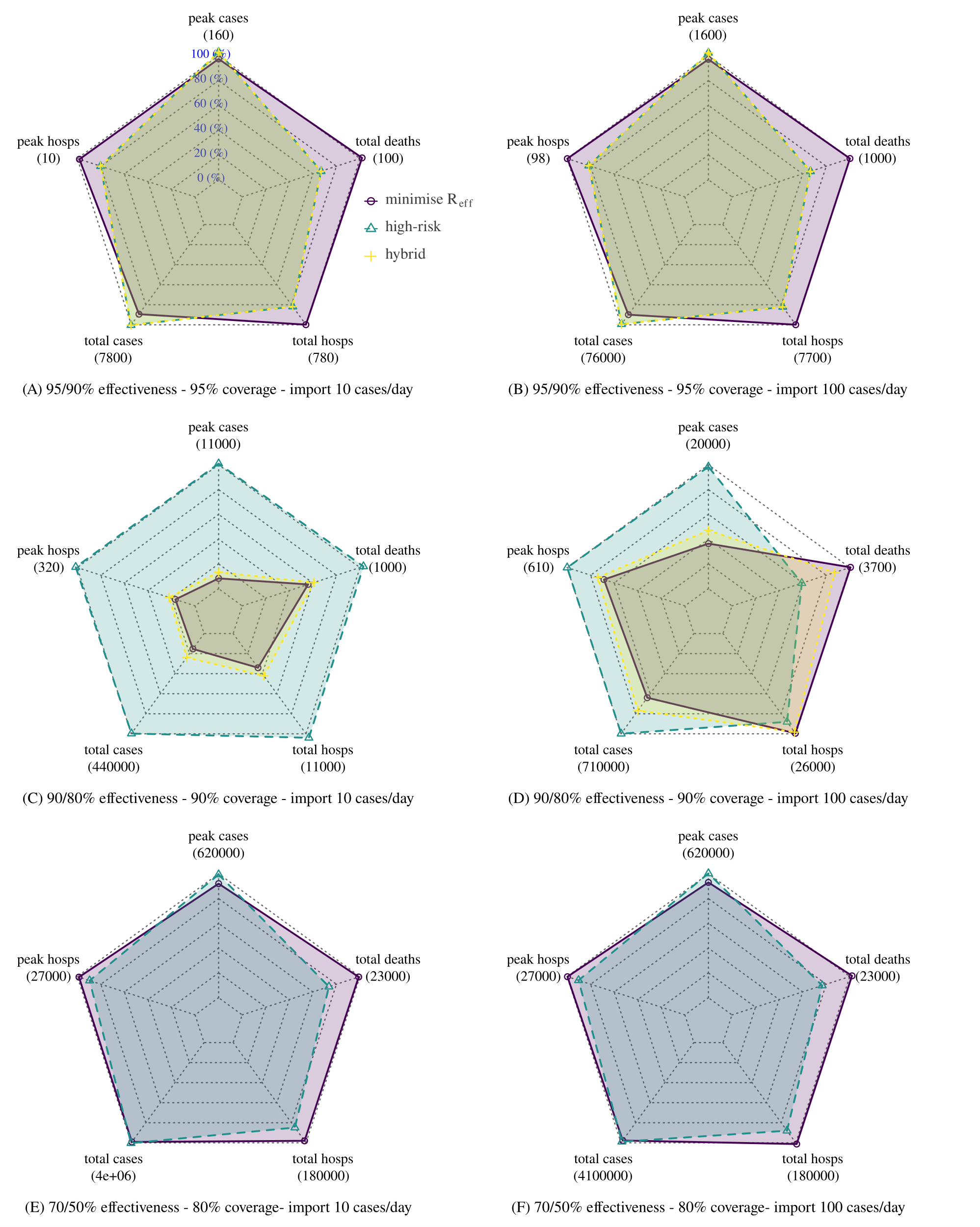


**Figure S8: Varying daily number of imported cases – the changes of outcome rankings among vaccine strategies (spread-minimising, high-risk, and hybrid strategies) (R_0_=4·5)**

Note: Forecasts for a two-year simulation with three vaccine strategies under three vaccine scenarios with varying daily number of imported cases that blend into community. The hybrid vaccine strategy is not available in the last two scenarios as the total uptake is not enough for the herd immunity threshold. The “total cases” measure only counts community cases, which excludes 7,300 imported cases for 10 imported cases/day or 73,000 imported cases for 100 imported cases/day.


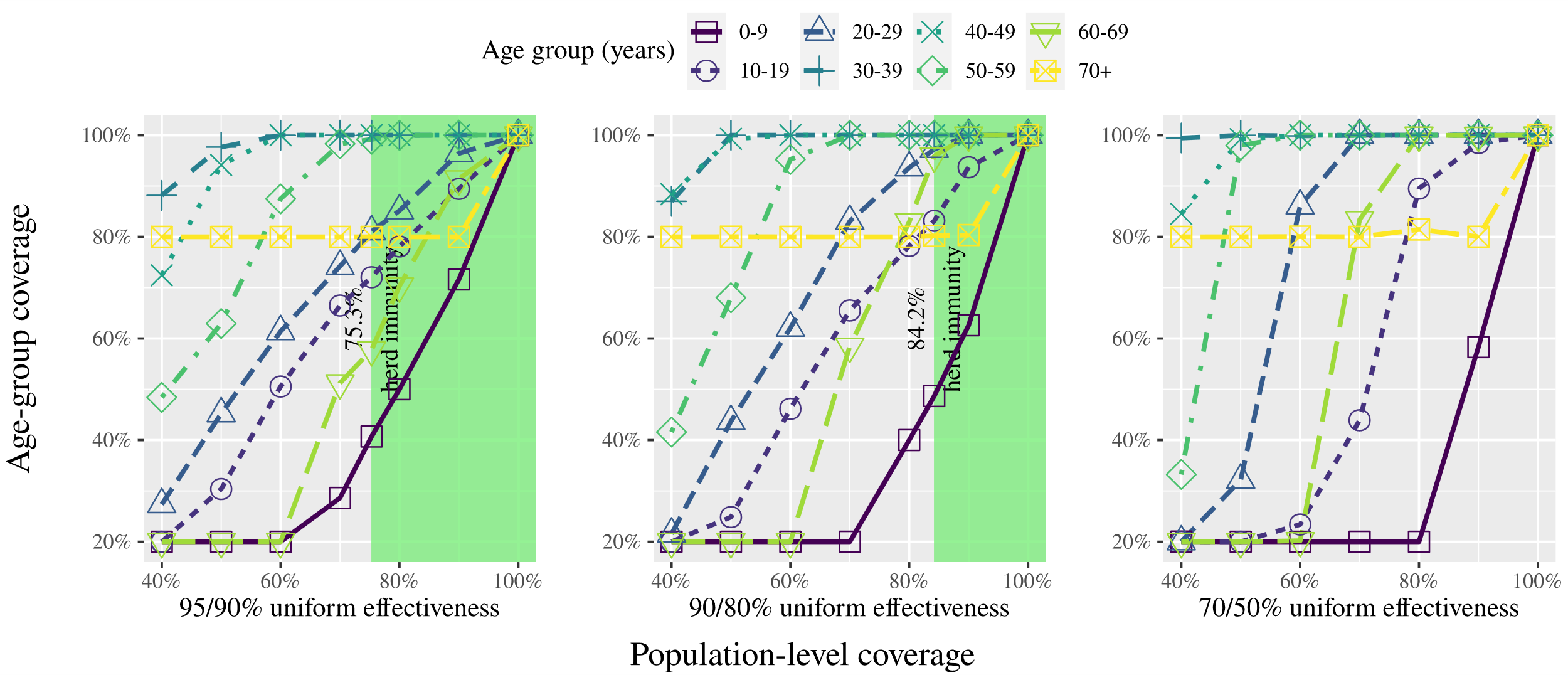


**Figure S9: Vaccine allocations of the spread-minimising strategy and minimal herd-immunity uptake levels customised for the combined Māori and Pasifika populations**

**Appendix S1 – The age-stratified SEIR model**

We used an age-stratified SEIR model with a presymptomatic infectious phase. Supplemental Figure S1 illustrates the age-stratified extended SEIR model for one age group *i*. Compartment *E_i_* corresponds to exposed individuals, *E_i_^v^* to exposed vaccinated individuals, *P_i_* to presymptomatic infectious cases, *Id_i_* to post-symptomatic clinical cases, *Is_i_* to asymptomatic/paucisymptomatic (subclinical) infected cases, and *R_i_* to recovered individuals of age group *i*. Compartment *D* includes all deaths from COVID-19. *P_i_* and *Id_i_* are associated with “clinical cases” that will develop (*P_i_*) or have developed (*Id_i_*) clinically-detectable features of disease, i.e. moderate to severe symptoms. *Is_i_* includes subclinical cases that are either asymptomatic or paucisymptomatic. The susceptible compartment (*S_i_*) includes people of age group *i* without vaccination. The vaccinated compartment (*V_i_*) refers to vaccinated people in age group *i*. Parameters used in this model are listed in Supplemental Table S1.

**The Next Generation Matrix**

Based on the contact matrix of New Zealand (NZ)^17,18^ where each individual of age group *i* makes contact with *c_ij_* individuals of age group *j*, we derived the force of infection for an individual in age group *i* is:

|  | $\lambda_{i}=U_{i}\sum_{j} c_{ij}(P_{j}+{Id}_{j}+{fIs}_{j})/N_{j}$ | (1) |
| --- | --- | --- |

where *U_i_* is the susceptibility of an age group *i*, *Id_j_*, *Is_j_* and *P_j_* are the clinically symptomatic, subclinical and presymptomatic cases in age group *j* respectively. *N_j_* is the population size of age group *j*. Therefore, $(P_{j}+{Id}_{j}+{fIs}_{j})/N_{j}$ is equivalent to the probability of encountering an infectious case per contact.

An entry at row *i* and column *j* of the next generation matrix (NGM) of unvaccinated population for the basic reproduction number R_0_ considering infected cases for each age group is as follows:

|  | ${NGM}_{ij}^{0}=U_{0}\frac{u_{i}N_{i}c_{ij}}{N_{j}}\left( \rho_{j}\left( t_{P}+t_{d} \right)+f\left( 1-\rho_{j} \right)t_{S} \right)$, | (2) |
| --- | --- | --- |

where $t_{P}=1/\delta$, $t_{d}=1/\gamma_{d}$ are presymptomatic and symptomatic infectious periods for clinical infections; $t_{s}=1/\gamma_{s}$ is the infectious period of subclinical cases; $f$ is an assumed reduction of infectiousness in subclinical cases compared with clinical ones; *N_j_* is the population size of an age group *j*; 𝜌*_j_* is the relative clinical fraction for age group *j*; and *U_0_* and *u_i_* are respectively the susceptibility scaling factor and relative susceptibility of age group *i* so the absolute susceptibility of the age group *i* is *U_i_=U_0_u_i_*. The initial value of susceptible group *S_i_* is *N_i_*, which is the population of age group *i*. The leading eigen value of the NGM^0^ is the basic reproduction number R_0_.

By calculating the unscaled NGM (without *U_0_*), where each entry is $unscaled\_{NGM}_{ij}^{0}={NGM}_{ij}^{0}/U_{0}$, we can infer the value of *U_0_* given an assumed R_0_ value (assumed R_0_=2·5) as:

|  | $U_{0}=R_{0}/lead\_eigen(u{nscaled\_NGM}^{0})$, | (3) |
| --- | --- | --- |

where $lead\_eigen(u{nscaled\_NGM}^{0})$ is the leading eigen value of the unscaled NGM. For effective reproduction number (R_eff_), an element of the initial NGM can be simplified as follows:

|  | ${NGM}_{ij}^{v}=\frac{U_{0}u_{i}\left( N_{i}-V_{i}e_{i} \right)c_{ij}}{N_{j}}\left( \rho_{j}^{'}\left( t_{P}+t_{d} \right)+f\left( 1-\rho_{j}^{'} \right)t_{S} \right)$, | (4) |
| --- | --- | --- |

where *V_i_* is the vaccinated compartment of age group *i* and $\rho_{j}^{'}=\rho_{j}\frac{N_{i}-V_{i}e_{d}}{N_{i}-V_{i}e_{i}}$ is the transformed clinical rate for both vaccinated and unvaccinated cases*.*

The simulation of different vaccine strategies can evaluate the outcomes of the vaccine allocations without other effects, such as the reinforcement of the vaccination process and the waning vaccine effect. This enables analyses of how the distribution of immunisation influences the outcomes on the medical system and total deaths. The transition from infections with clinical disease (*Id_i_*) to recovery and deaths is elaborated into an intermediate compartment *H_i_* (Supplemental Figure S2), which includes the hospitalised cases of age group *i*.

All these values to be minimised in vaccine strategies are initial values and are expected to be reduced as a result of increasing immunity due to viral spread among the community and the continuous vaccination process.

## Appendix S2 – Comparison of vaccination strategies

This supplementary material provides the forecasted outcomes from two-year simulations of vaccination strategies investigated in the study, which include: (1) the spread-minimising strategy that minimises R_eff_; (2) the high-risk targeting strategy that prioritises the oldest population, i.e. the population with the highest risk for COVID-19 disease and deaths; and (3) the hybrid strategy that minimises R_eff_ to achieve the herd immunity threshold (HIT) (R_eff_≤1, if possible) using the least vaccine coverage and prioritises the rest of the vaccine uptake on the oldest population. The third strategy is only available when the first strategy can achieve HIT. Supplemental Figure S3 illustrates the results of vaccine distributions by the spread-minimising strategy (strategy 1).

The standard approach to vaccination in areas of active disease transmission is to prioritise groups of high-risk of poor outcomes, such as older age groups.^19^ When implementing a herd-immunity strategy, it is also important to eliminate the virus as quickly as practicable. Thus, a third vaccine strategy, called ‘hybrid strategy’, is to reduce R_eff_ to 1 before prioritising the high-risk population. This strategy aims to balance between two potential risks: the spreading rate if SARS-CoV-2 is introduced before completing the vaccination process and the total COVID-19-related deaths and hospitalisations.

In the open border simulation, the number of total imported cases (that blend into the community) is 7,300 cases. With a fixed age distribution of the past arrived cases,^14^ the predicted hospitalised and death cases from the 7,300 imported cases are constant across all scenarios and vaccine strategies. The total number of hospitalised cases is:

$H_{total}=\sum_{i} {imports}_{i}*{hosp\_rate}_{i}*7300$,

and the total number of death cases is:

$D_{total}=\sum_{i} {imports}_{i}*{death\_rate}_{i}*7300$,

where *imports_i_* is the number of daily imported cases that fall in the age group *i*, *hosp_rate_i_* and *death_rate_i_* are the hospitalisation and death rate of the age group *i* respectively. We assumed all imported cases are unvaccinated before infections. Thus, their age-stratified death rates and hospitalisation follow the age-stratified death rates modelled by Verity et al.^12^ and the age-stratified hospitalisation rates from Episurv^14^ respectively. For the course of two years (730 days), the expected total hospitalisations of imported cases are 444 cases and the expected total deaths of imported cases are 49·6 cases.

The forecasted results of infected cases, hospitalised cases, and death cases of three vaccine strategies in various vaccine scenarios and R_0_ values are shown in Supplemental Tables S2–9. Supplemental Table S2 includes the scenarios of R_0_=4·5 and vaccines with uniform effectiveness across age groups where vaccine is allowed for people aged over 16. This table complements Table 1 and 2 in the main text that show the modelling results of the same R_0_ value but vaccination is allowed for different age groups. Similarly, Supplemental Tables S3–5 contain the modelling results of the same vaccine scenarios but a lower R_0_ value of 6, while Tables S6-8 are for the R_0_ value of 2·5. Supplemental Table S9 shows the modelling results of vaccines with immune senescence. Results are rounded to the third significant number or rounded to integers if smaller than 100. Although there is high coverage with 95% vaccine effectiveness, the number of cases might be still high. However, the percentages of hospitalised cases have been reduced. Supplemental Figure S4 shows the changes of active cases and hospitalisations over the simulation period when the vaccine scenario is 95/70% uniform effectiveness and 80% coverage for total uptake. Since there are continuous introductions of oversea cases, these measures are not reduced to (near) 0(s) after reaching their peaks.

## Appendix S3 – Minimal herd immunity requirements for different R_0_ values, vaccine effectiveness, and vaccination strategies

This section provides the additional analyses of HIT requirements regarding vaccine effectiveness in many scenarios of different R_0_ values, vaccination age restriction, and vaccine strategies. The analyses of HIT requirements where vaccination is allowed for all age groups are available in the main text. Where R_0_ is in the range of [2, 3·5], maximum vaccine coverage for each age is assumed to be 90%. With higher R_0_ values (>4), we enabled the maximum vaccine coverage to as much as 100% (no limit). HIT is not achievable for higher R_0_ values when vaccination is restricted to the 12 or 16 year-and-older. Figure S5-6 show the HIT requirements for lower R_0_ values (no greater than 3·5). Without vaccinating the children aged under 12, HIT is not achievable for R_0_=3·5.

**Appendix S4 –** **Sensitivity Analysis**

The limitation of this study was that the contact matrix were obtained through synthesising based on population demographics and residential statistics in.^17^ Contact patterns are also subject to variation at different time of the year. To address this limitation, we analysed the changes in vaccine allocations and modelling results by adding a random value to each element *c_ij_* of the contact matrix. The added random value for each *c_ij_* was generated by a uniform distribution with the mean of 0 and the boundaries of $(-10\%c_{ij},10\%c_{ij})$. We generated 50 new contact matrices using this method. Supplemental Figure S7 provides the vaccine allocations of corresponding vaccine scenarios with the original (on the right) and a noise-added contact matrix (on the left). The variations of vaccine allocations of the spread-minimising strategy were smaller than the variations by different random seeds. The two-year modelling results of 50 different noise-added contact matrices had small standard deviations compared with mean values (Supplemental Table S9).

Higher numbers of imported cases will normally result in higher numbers of infections, hospitalisations, and deaths. This was confirmed by varying the number imported cases and observing the outcomes in different scenarios (Supplemental Figure S8). The pairs of Figures S8A vs. S8B, S8C vs. S8D, and S8E vs. S8F show the same scenario with different numbers of daily imported cases. The rankings of strategies in terms of cases and peak hospitalisations remained unchanged when the number of daily imported cases increases. However, the total hospitalisations and deaths of the high-risk targeting strategy was the lowest among three strategies in the cases where the uptake was low and the vaccine effectiveness (VE) of infection reduction was high, e.g. 90/80% effectiveness and 90% coverage (Figures S8C and S8D). This is due to, under the high-risk targeting strategy, HIT being achieved faster through a higher number of imported cases. It is noted that the higher peak of hospitalisations of the high-risk targeting strategy (324), which is near the hospitalisation capacity,^20^ could result in additional deaths from non-COVID-19 causes that are not able to receive appropriate and timely treatment. Only in the scenario of 70/50% effectiveness and 80% coverage in Figures S8E and S8F, where the R_eff_ value is much higher than 1 (no HIT), the outcomes between two different numbers of imported cases are similar. This is because, when R_eff_ >> 1, the total number of imported cases is very small compared with the outbreak size.

**Appendix S5 – Modelling Māori and Pasifika populations**

This section provides modelling results for Māori and Pasifika populations using the age-group distribution of their combined populations. The vaccine allocations of the high-risk targeting strategy priorities maximum coverage for the oldest age groups before allocating younger groups. For Māori and Pasifika populations, this strategy can cover up to younger groups compared with its coverage for the whole NZ when the proportions of total vaccinated population are the same. This is due to that Māori and Pasifika populations have a much lower population distribution on old age groups. Supplemental Figure S9 illustrates the vaccine allocations of the spread-minimising strategy for the combined Māori and Pasifika populations. The age-group prioritisation of the spread-minimising strategy in these populations is analogous to overall NZ population with 30–49 year olds to be prioritised. However, the strategy of minimising R_eff_ for the Māori and Pasifika populations allocates more vaccinations to the older age group (50–69 year olds) compared with the same strategy for the whole NZ. The minimal uptake for HIT is also lower in these populations. A possible reason is that the Māori and Pasifika populations are relatively young populations^9^ with a larger proportion of the population in the youngest age group (0–9 years old), which does not contribute as much to viral spread. This age group has the lowest susceptibility to the virus and does not have as many contacts compared with other groups (e.g. 30–49 years).

Two-year simulations of various scenarios were run for these populations where the transmission from other ethnic groups was considered as imported cases. These results as shown in Supplemental Table S11-12 have similar trends as described for whole NZ population but with smaller scales of all investigated measures. The differences among the measures of the vaccine strategies, especially at 80% coverage, are much smaller. The reason is that, with 80% coverage, the vaccine distributions only differ substantially in the age group 0–9 (with the largest population), which does not contribute much to both viral spread, hospitalisations, and deaths.

## References

1. Riou J, Althaus CL. Pattern of early human-to-human transmission of Wuhan 2019-nCoV. *bioRxiv* 2020: 2020.01.23.917351.

2. Wu JT, Leung K, Leung GM. Nowcasting and forecasting the potential domestic and international spread of the 2019-nCoV outbreak originating in Wuhan, China: a modelling study. *Lancet* 2020; **395**(10225): 689–97.

3. Li Q, Guan X, Wu P, Wang X, Zhou L, Tong Y, et al. Early Transmission Dynamics in Wuhan, China, of Novel Coronavirus–Infected Pneumonia. *N Engl J Med* 2020; **382**(13): 1199–207.

4. Rothe C, Schunk M, Sothmann P, Bretzel G, Froeschl G, Wallrauch C, et al. Transmission of 2019-nCoV Infection from an Asymptomatic Contact in Germany. *N Engl J Med* 2020; **382**(10): 970–1.

5. Byrne AW, McEvoy D, Collins A, Hunt K, Casey M, Barber A, et al. Inferred duration of infectious period of SARS-CoV-2: rapid scoping review and analysis of available evidence for asymptomatic and symptomatic COVID-19 cases. *medRxiv* 2020: 2020.04.25.20079889.

6. McAloon C, Collins Á, Hunt K, Barber A, Byrne AW, Butler F, et al. Incubation period of COVID-19: a rapid systematic review and meta-analysis of observational research. *BMJ Open* 2020; **10**(8): e039652.

7. Casey M, Griffin J, McAloon CG, Byrne AW, Madden JM, McEvoy D, et al. Pre-symptomatic transmission of SARS-CoV-2 infection: a secondary analysis using published data. *medRxiv* 2020: 2020.05.08.20094870.

8. Jefferies S, French N, Gilkison C, Graham G, Hope V, Marshall J, et al. COVID-19 in New Zealand and the impact of the national response: a descriptive epidemiological study. *Lancet Public Health* 2020; **5**(11): e612–e23.

9. Stats NZ Tatauranga Aotearoa. New Zealand Population Estimates. 2020.

10. Davies NG, Klepac P, Liu Y, Prem K, Jit M, Eggo RM. Age-dependent effects in the transmission and control of COVID-19 epidemics. *Nat Med* 2020; **26**(8): 1205–11.

11. Davies NG, Kucharski AJ, Eggo RM, Gimma A, Edmunds WJ, Jombart T, et al. Effects of non-pharmaceutical interventions on COVID-19 cases, deaths, and demand for hospital services in the UK: a modelling study. *Lancet Public Health* 2020; **5**(7): e375–e85.

12. Verity R, Okell LC, Dorigatti I, Winskill P, Whittaker C, Imai N, et al. Estimates of the severity of coronavirus disease 2019: a model-based analysis. *Lancet Infect Dis* 2020; **20**(6): 669–77.

13. Steyn N, Binny RN, Hannah K, Hendy SC, James A, Kukutai T, et al. Estimated inequities in COVID-19 infection fatality rates by ethnicity for Aotearoa New Zealand. *N Z Med J* 2020; **133**(1521): 12.

14. The Institute of Environmental Science and Research. EpiSurv, New Zealand notifiable disease surveillance database. <https://surv.esr.cri.nz/episurv/> (accessed 11 November 2020).

15. Steyn N, Binny RN, Hannah K, Hendy SC, James A, Lustig A, et al. Māori and Pacific People in New Zealand have higher risk of hospitalisation for COVID-19. *medRxiv* 2020: 2020.12.25.20248427.

16. Ministry of Health New Zealand. COVID-19: Vaccines. 2021. <https://www.health.govt.nz/our-work/diseases-and-conditions/covid-19-novel-coronavirus/covid-19-vaccines> (accessed 27 April 2021).

17. Prem K, van Zandvoort K, Klepac P, Eggo RM, Davies NG, Cook AR, et al. Projecting contact matrices in 177 geographical regions: an update and comparison with empirical data for the COVID-19 era. *medRxiv* 2020: 2020.07.22.20159772.

18. Prem K, Cook AR, Jit M. Projecting social contact matrices in 152 countries using contact surveys and demographic data. *PLoS Comput Biol* 2017; **13**(9): e1005697.

19. Department of Health and Social Care GOV.UK. Priority groups for coronavirus (COVID-19) vaccination: advice from the JCVI, 30 December 2020, 2020.

20. Ministry of Health New Zealand. Ventilators and ICU bed capacity, 2020.
